# HLA-Shuttle: A system for enhancing antigen presentation in immunologically cold tumors

**DOI:** 10.1126/sciadv.aeb0821

**Published:** 2026-01-01

**Authors:** Daniel Hwang, Molly C. Erdman, Santosh Adhikari, Ram Pantula, Kaya Epstein, Peiyao Li, Francesca Costabile, Photis Rotsides, Chloe S. Wang, Shirley M. Sun, Hossein Fazelinia, Lynn A. Spruce, Tim Fugmann, Trendelina Rrustemi, Wai Tuck Soh, Alvin Farrel, Muzamil Y. Want, Andrea Facciabene, Mustafa Mir, John M. Maris, Nikolaos G. Sgourakis

**Affiliations:** ^1^ Center for Computational and Genomic Medicine, Department of Pathology and Laboratory Medicine, Children’s Hospital of Philadelphia, Philadelphia, PA 19104, USA.; ^2^ Department of Biochemistry and Biophysics, Perelman School of Medicine, University of Pennsylvania, Philadelphia, PA 19104, USA.; ^3^ Department of Cell and Developmental Biology, Perelman School of Medicine, University of Pennsylvania, Philadelphia, PA 19104, USA.; ^4^ Howard Hughes Medical Institute and Children’s Hospital of Philadelphia, Philadelphia, PA 19104, USA.; ^5^ Epigenetics Institute, Perelman School of Medicine, University of Pennsylvania, Philadelphia, PA 19104, USA.; ^6^ Cancer Biology Graduate Group, Perelman School of Medicine, University of Pennsylvania, Philadelphia, PA 19104, USA.; ^7^ Division of Oncology and Center for Childhood Cancer Research, Children’s Hospital of Philadelphia, Philadelphia, PA 19104, USA.; ^8^ Department of Radiation Oncology, University of Pennsylvania, Philadelphia, PA 19104, USA.; ^9^ Immunology Graduate Program, Perelman School of Medicine, University of Pennsylvania, Philadelphia, PA 19104, USA.; ^10^ Proteomics Core Facility, Children’s Hospital of Philadelphia, Philadelphia, PA 19104, USA.; ^11^ Alithea Biotechnology, Freiburg im Breisgau, Germany.

## Abstract

Epitopic peptides presented by class-I human leukocyte antigen (HLA-I) proteins provide the basis of immune surveillance by T cells. Conversely, reduced surface HLA-I expression is a hallmark of immune evasion by latent viral infections and cancer, which confounds the identification of peptide antigens and neoantigens. Here, we outline a system (HLA-Shuttle) for in vitro manipulation of cells with engineered components of the HLA-I processing machinery to confer a continuum of chaperoning activity throughout their trafficking pathway. HLA-Shuttle restores antigen presentation in immunologically cold neuroblastoma cells, enabling identification of multiple tumor-associated antigens with therapeutic potential. Cellular trafficking assays and single-particle tracking reveal a global stabilization of HLA-I molecules, extension of their cell-surface lifetime and microdomain formation. HLA-Shuttle can be used across a range of aberrant cellular states where low antigen expression remains a bottleneck for the identification of endogenous peptide antigens.

## Teaser

HLA-Shuttle enhances production of HLA class I molecules in neuroblastoma, leading to the discovery of tumor-associated antigens.

## INTRODUCTION

A prominent immune evasion strategy used by cancers is the down-regulation of class-I human leukocyte antigen (HLA-I), resulting in a failure for surveilling cytotoxic T lymphocytes (CTLs) to recognize and eliminate aberrant cells (*1*). This class of cancers, termed immunologically “cold” tumors, are characterized by low levels of immune cell infiltration with limited signs of inflammation and immune activity (*2*). This immunological quiescence is often achieved through the dysregulation of the antigen processing and presentation (APP) pathway (*1*), which can dramatically reduce tumor-associated antigen (TAA) and neoantigen presentation by HLA-I (*3*). The low antigenicity of cold tumors imposes considerable roadblocks to immunotherapy development and efficacy. Low HLA-I has multiple negative implications for immunotherapy outcomes, both stymieing in vivo efficacy, while also hindering identification of targetable peptides antigens. This issue is a hallmark of neuroblastoma, a common childhood malignancy that accounts for 15% of pediatric cancer–related deaths (*4*). Neuroblastomas are notoriously nonimmunogenic tumors (*5*), characterized by reduced HLA-I and -II expression and low mutational burden, which challenges the identification of appropriate antigen targets. Moreover, despite recent progress (*6*), the scarcity of tumor-infiltrating lymphocytes (TILs) or TAA-specific T cells hinders target identification through evaluating TIL responses to candidate peptides. However, recent approaches combining transcriptomics with mass spectrometry (MS)–based immunopeptidomics (*7*) have enabled identification of pHLA-I targets with therapeutically relevant features for CAR-T development (*8*, *9*). Nevertheless, immunopeptidomic approaches are still challenging because of the low abundance of HLA-presented peptides on the cell surface confounding their capture and identification (*10*). These issues are compounded in neuroblastoma and other cancers with low HLA expression like small cell lung cancer (*5*). Given the high sensitivity of T cells for their cognate antigens (*11*), it is probable that many promising therapeutic targets may escape detection, further contributing to the paucity of targetable biomarkers in neuroblastoma.

To address this issue, we have expanded the function of the primary HLA-I chaperone tapasin, a key component of the peptide loading complex (PLC) (*12*). Tapasin has multiple key functions that promote the production of peptide-loaded HLA-I (pHLA-I) molecules including: (i) maintaining a pool of folded, peptide-receptive HLA-I molecules; (ii) recruiting nascent HLA-I molecules to the TAP transporter, which translocates peptides into the endoplasmic reticulum (ER) for loading on HLA-I; and (iii) promoting the selection of a repertoire of optimal peptide ligands for prolonged trafficking and display on the cell surface [peptide optimization, or editing (*12*)]. As a consequence of its essential role in APP, tapasin expression is often disrupted in cancers, (*13*), with cells lacking tapasin having up to 90% reduction in surface HLA-I (*14*). These features of tapasin expression in normal cells and in cancers led us to hypothesize that tapasin may be a useful agent for enhancing APP function in cold tumors. We have developed an engineered variant, termed tapasin-TM that restores antigen presentation in neuroblastoma cell lines and stabilizes HLA-I complexes, enabling identification of additional TAA’s.

## RESULTS

### Down-regulation of HLA-I antigen processing machinery is a hallmark of cancer immune evasion

**Fig. 1.**
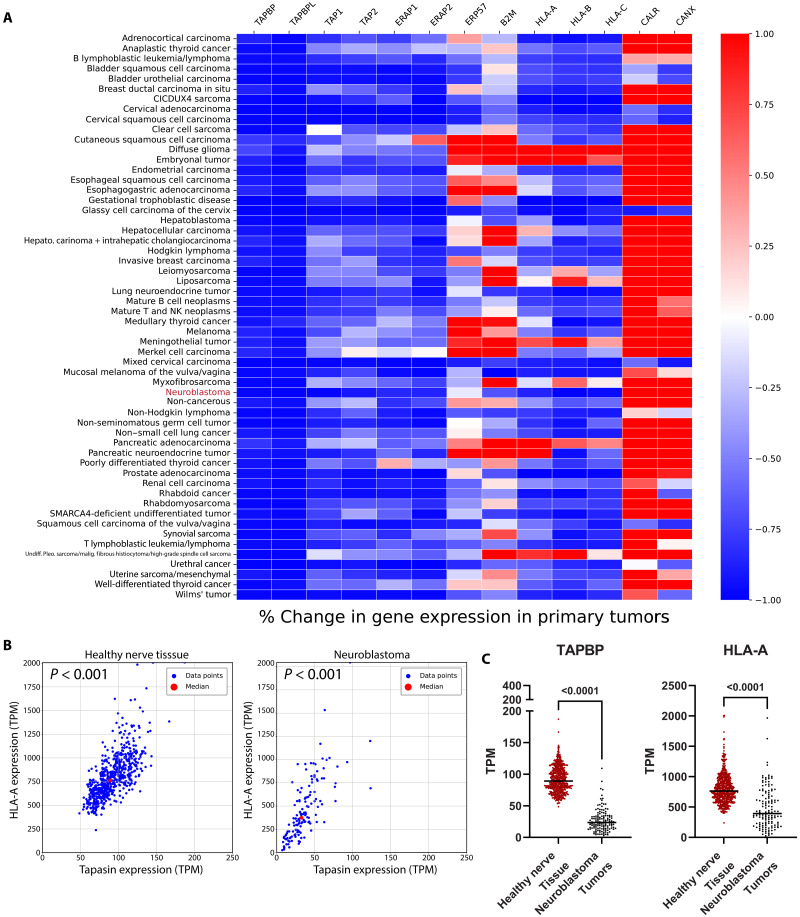
Tap-binding protein (TAPBP, tapasin) expression is down-regulated across cancers. (**A**) Change in HLA-A expression between healthy [GTex V8 (*37*) mean transcripts per kilobase million, (TPM)] and cancerous [The Cancer Dependency Map Project (DepMap (*76*) Omics Batch Corrected TPM]. (**B**) Expression of HLA-A versus tapasin in healthy tibial nerve tissue (left) and neuroblastoma cell lines (right). Statistics were performed by Pearson correlation analysis. (**C**) Tapasin and HLA-A expression in TPM for healthy tibial nerve and neuroblastoma tumors is shown. Line represents median. Statistics performed by unpaired two-tailed *t* test.

We evaluated the expression of key APP genes between normal and cancerous tissues. We matched RNA expression data from The Cancer Dependency Map Project [(*15*)] by presumed tissue of origin with healthy tissue data from GTex (Fig. 1A). Most APP genes were down-regulated in cancers, excluding the cochaperones calreticulin and calnexin, which participate in cancer pathology (*16*). Notably, HLA-A*, B*, and C* allotype expression was not uniform across cancers, while HLA-I chaperones tapasin [tap-binding protein (TAPBP)] and TAPBPR were universally down-regulated across cancers. Extending our analysis to neuroblastoma tumors, we found that both HLA-A and tapasin were significantly lower in expression by comparison to healthy tissues (Fig. 1, B and C). Thus, diminished expression of APP genes, especially tapasin, is a hallmark of cancer immune evasion, corroborating previous studies (*13*).

### Engineered Tapasin-TM increases presentation of folded HLA-I complexes in cold neuroblastoma cells

**Fig. 2.**
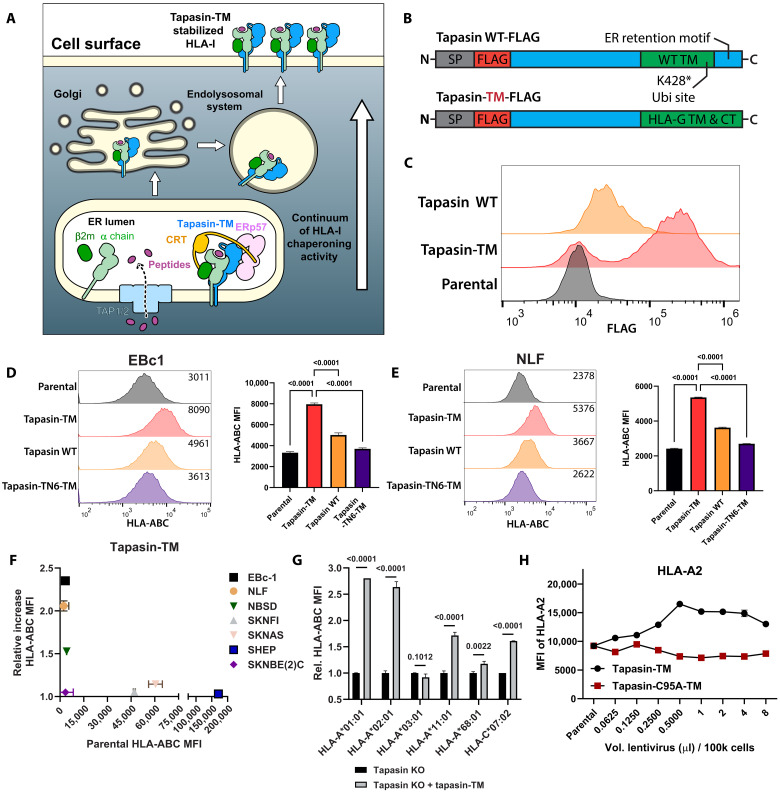
Engineered tapasin-TM restores HLA-I surface expression in immunologically cold neuroblastoma cells. (**A**) Schematic depicting tapasin-TM trafficking to the cell surface. (**B**) Schematic depicting differences between tapasin WT and tapasin-TM constructs. Tapasin WT contains the C-terminal ER retention motif and lysine-428 residue (UniProt: O15533), which are replaced in tapasin-TM. (**C**) Expression of tapasin constructs via intracellular staining for FLAG in EBc1 cells. (**D** and **E**) Neuroblastoma cell lines were transduced with a titration of lentivirus and stained for HLA-I complex using W6/32 antibody (*77*). (D) HLA-I expression and quantification of median fluorescence intensity (MFI) for EBc1 and (E) NLF cells transduced with tapasin variants. (**F**) Relative increase in HLA-I by W6/32 staining for tested neuroblastoma lines. Negative values were reported as one. Two to four technical replicates pooled from two independent experiments. Significance for (D) and (E) was determined by one-way analysis of variance (ANOVA) followed by multiple comparisons testing of each condition versus the tapasin-TM transduced line. (**G**) The tapasin gene was knocked out in 721.221 cells expressing a single HLA-I allele (monoallelic) via electroporation of Cas9/sgRNA RNPs before transduction with lentivirus encoding tapasin-TM or an empty vector lentivirus. Change in HLA-I MFI was determined as the ratio between cells transduced with tapasin-TM and those transduced with the empty vector. Significance was determined by two-way ANOVA followed by Tukey’s multiple comparisons test. (**H**) EBc1 cells were transduced with either tapasin-TM or tapasin-C95A-TM, which abrogates interactions with the Erp57 thiolreductase, and then evaluated for surface HLA-A2 expression.

Given the widespread down-regulation of tapasin across cancers, we sought to test whether exogenous tapasin expression may restore antigen presentation in cold tumor cells. However, levels of tapasin are regulated not only at the transcriptional level but also at the protein level, which could be exploited by cancer cells to limit the effectiveness of a tapasin-based APP adjuvant. To address this possibility, we replaced the C-terminal transmembrane and cytosolic domain of wild-type (WT) tapasin with the analogous domain from HLA-G*, an oligomorphic type 1b HLA protein, enabling trafficking of tapasin throughout the secretory pathway, including the ER, Golgi, and cell surface, by removal of the native tapasin ER retention motif (*17*). This approach was previously implemented by our group for TAPBPR, a tapasin homolog, leading to widespread distribution of the chaperone in the ET, Golgi, and at the cell surface (*18*). Replacement of the native tapasin domains also eliminates K428, a ubiquitination site that targets tapasin for degradation (*19*) (Fig. 2, A and B). This variant, termed tapasin-TM, showed higher expression than WT tapasin in the cold neuroblastoma cell line EBc1 and in all tested lines (Fig. 2C and fig. S2A). Moreover, as expected, tapasin-TM trafficked to the cell surface, as detected by an N-terminal FLAG-tag (fig. S3). We tested the effects of WT tapasin, tapasin-TM, and tapasin-TN6-TM [the TN6 mutant cannot chaperone HLA-I molecules in vitro (*20*)], on expression levels of folded surface HLA-I molecules in seven neuroblastoma cell lines with varying degrees of baseline HLA-I expression by staining with the pan-allelic antibody W6/32. Among tested cell lines, EBc1, NLF, and NBSD cells showed marked HLA-I up-regulation upon lentiviral transduction of tapasin-TM (Fig. 2, D to F, and fig. S1, A to E). This effect appeared to depend on baseline HLA-I expression, with three of four cell lines with very low HLA-I expression showing prominent up-regulation (Fig. 2F). Moreover, using the allotype-specific BB7.2 antibody (*21*), we found similar up-regulation of the HLA-A*02:01 allomorph (HLA-A2) expression in EBc1 and NLF cells (fig. S2B). We also tested whether TAPBPR, a related auxiliary chaperone with a quality control role in the HLA-I APP (*22*) that can promote peptide exchange in vitro (*23*), could affect surface HLA-I levels. Expression of TAPBPR-TM (*18*), which has the same C-terminal domain replacement as tapasin-TM, had a negative effect on overall surface HLA-I (fig. S2C), suggesting a retention of nascent molecules that is consistent with its role as an major histocompatibility class I quality control chaperone (*12*, *24*).

HLA-I molecules are highly polymorphic, showing a range of dependencies on tapasin for peptide loading and cell-surface expression (*25*, *26*). To evaluate whether tapasin-TM could enhance production of individual HLA-I allotypes, we used 721.221 lymphoblastoid cells, which are natively HLA-I KO, but complemented with individual HLA-I alleles as transgenes (*27*). Using CRISPR-Cas9, we knocked out the tapasin gene in 721.221 cells and evaluated the effects of tapasin-TM on 6 HLA-A* and 1 HLA-C* alleles, spanning previously reported levels of tapasin dependency (*26*). For most monoallelic cell lines, tapasin-TM expression resulted in significant benefit to HLA-I production (Fig. 2G and figs. S1G and S4). Notably, although the tapasin-dependent allele HLA-A*01:01 displayed the highest degree of benefit from coexpression with tapasin-TM, HLA-A*02:01–expressing cells benefited at a similar level, in alignment with our data in HLA-A2^+^ neuroblastoma cell lines. In contrast, HLA-A*03:01 and HLA-*68:01, which are known tapasin-independent alleles (*26*), showed minimal effects upon transduction with tapasin-TM. These results suggest that our system leveraging Tapasin-TM, HLA-Shuttle, can restore production of HLA-I in cells with severe defects to this pathway, and that these effects are generalizable across most common HLA-I alleles that depend on tapasin for surface expression.

Tapasin stability and therefore efficient PLC assembly is dependent on the thiolreductase ERp57, which forms a disulfide bond with tapasin at C95 (*28*). To establish whether the effects of tapasin-TM on increased surface HLA-I were dependent on ERp57, we expressed the sulfhydryl-deficient tapasin-C95A-TM mutant in EBc1 cells. Notably, the C95A mutation abrogated tapasin-TM–mediated up-regulation of HLA-I (Fig. 2H and fig. S1H) and led to reduced levels of tapasin-C95A-TM within cells (fig. S1I). We attempted to assess whether ERp57 cotrafficked with tapasin to the cell surface by flow cytometry but could not detect ERp57 on the surface, while ERp57 was detectable intracellularly (fig. S1J). Together, these results suggest that tapasin-TM relies on interactions with the cochaperone ERp57 to exert its effects on HLA-I molecules. Moreover, these data imply that the intracellular functions of tapasin-TM are necessary for its activity.

### Tapasin-TM globally stabilizes HLA-I molecules across neuroblastoma lines and HLA-I allotypes

**Fig. 3.**
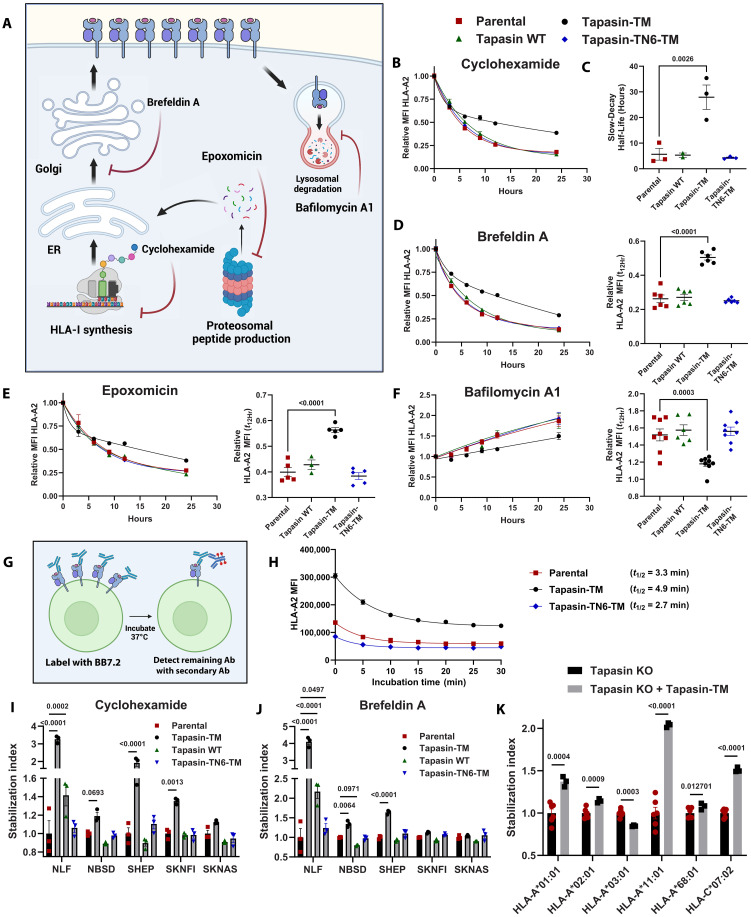
Tapasin-TM stabilizes surface HLA-I complexes across neuroblastoma lines and HLA-I alleles. (**A** to **F**) Ebc1 parental or tapasin variant transduced cells were treated with cycloheximide, brefeldin A, bafilomycin A1, epoxomicin, or vehicle and analyzed for surface HLA-A2 expression (BB7.2 antibody staining). Data normalized to cells treated with vehicle for 24 hours. For cycloheximide, brefeldin A, and epoxomicin, curves represent two-phase exponential decay regression. For bafilomycin A1, two-phase association regression is shown. Tapasin-TM cells represent tapasin-TM^Hi^ cells from figs. S3 and S4 (A) Schematic depicting action of each drug. Created in BioRender. Hwang, D. (2025) https://BioRender.com/odvmnxp. (B) Relative MFI HLA-A2 (*n* = 6 to 14 technical replicates compiled from two to three independent experiments) and (C) slow-decay half-life (derived from two to three independent experiments) for cells treated with cycloheximide. [(D) to (F)] Similar analysis for brefeldin A, epoxomicin, and bafilomycin A1–treated cells. Statistical significance for slow-decay half-life and MFI of HLA-A2 was determined by one-way ANOVA followed by Šidák multiple comparisons test. Each condition is compared to the parental cell line. (**G**) Antibody-based HLA-A2 internalization assay measuring anti–HLA-A2 antibody (BB7.2) internalization at 37°C. Created in BioRender. Hwang, D. (2025) https://BioRender.com/0vk9d6w. (**H**) MFI of HLA-A2 is shown. Half-life data are derived from one-phase decay regressions. (**I**) Stabilizing effect of tapasin variants across NB cell lines. NLF cells were treated for 8 hours; NBSD, SKNFI, and SKNAS for 18 hours; and SHEP treated for 48 hours with cycloheximide and (**J**) brefeldin A before staining with W6/32 antibody. Stabilization index was calculated by normalizing values to vehicle-treated controls followed by normalization to mean of the parental condition. Significance was determined by two-way ANOVA followed by multiple comparisons testing to parental cells. (**K**) Tapasin knockout HLA-I monoallelic 721.221 cells were complemented with tapasin-TM and HLA-I stability evaluated via brefeldin A treatment for 4 hours. Significance was determined by unpaired *t* test.

We next turned our attention to understanding how tapasin outside of the ER (extra-ER tapasin) may influence the kinetics and stability of HLA-I molecules. We hypothesized that outside the ER, tapasin-TM may confer additional stabilizing benefits globally, extending its ER function (*29*). To evaluate how surface tapasin-TM may influence HLA-I stability, we first separated tapasin-TM–expressing EBc1 cells according to their surface tapasin expression (fig. S3). We then performed a series of HLA-I assembly and trafficking assays to evaluate the influence of tapasin-TM on cell surface stability of HLA-A2 specifically, by staining with the BB7.2 antibody, which recognizes folded molecules (schematized in Fig. 3A). We first measured the decay of HLA-A2 molecules using the ribosomal inhibitor cycloheximide. All cells expressing tapasin-TM displayed slower decay kinetics when compared to WT tapasin or control cells (fig. S5A and Fig. 3B). We fitted two-phase decay curves to these data and found that tapasin-TM cells had a slow-decaying population with significantly longer half-life (Fig. 3C). Treatment with the ER-Golgi trafficking inhibitor brefeldin A or with the proteasomal inhibitor epoxomicin yielded similar results, leading to higher relative HLA-A2 expression during the treatment (Fig. 3, D and E, and fig. S5B). Moreover, treatment with lysosomal inhibitor bafilomycin A1 revealed that tapasin-TM–transduced cells had reduced sensitivity to bafilomycin A1 treatment (Fig. 3F and fig. S5C), implying reduced targeting to the lysosome. When comparing between surface tapasin-TM^Hi^ and tapasin-TM^Lo^ cells, surface tapasin-TM^Hi^ cells had more stable HLA-A2 molecules but also had somewhat lower surface HLA-A2 expression (fig. S5, D to F), suggesting that the stabilizing function of extra-ER tapasin and up-regulation of HLA-A2 may occur via different mechanisms. Nevertheless, the stabilizing effect of tapasin-TM was readily observable in the unnormalized flow cytometry data (fig. S6, A and B). We also investigated tapasin-TM–mediated stability enhancement of HLA-I at a higher temporal resolution. We measured the rate of internalization of BB7.2-labeled EBc1 cells and found reduced internalization rates in cells expressing tapasin-TM than parental or tapasin-TN6-TM–expressing cells over a 30-min time course (Fig. 3, G and H, and fig. S6C). These data establish that tapasin-TM has broad stabilizing effects on HLA-A2 molecules, resulting in longer cell surface half-life.

Next, to determine whether tapasin-TM–mediated stabilization would be applicable to other cell lines with varying degrees of HLA-I production, we used cycloheximide and brefeldin A treatment followed upon with W6/32 antibody labeling across five neuroblastoma lines. In NLF cells, tapasin-TM conferred an approximately threefold increase in stability of HLA-I molecules (Fig. 3, I and J, and fig. S6D). In alignment with our experiments evaluating HLA-A2 stability in EBc1 cells, nearly all cell lines tested displayed increase in HLA-I stability when expressing tapasin-TM, including cell lines that did not display enhanced HLA-I production, such as SKNFI (Figs. 3I and 2F). Last, to evaluate the HLA allelic dependence of this effect, we used our tapasin-KO 721.221 HLA-I monoallelic cell lines that were complemented with tapasin-TM. Following brefeldin A treatment, all tested HLA-I alleles showed enhanced stability in cells expressing tapasin-TM, except for HLA-A*68:01 and HLA-A*03:01(Fig. 3K), in agreement with our observed HLA-I up-regulation results (Fig. 2G).

### SMT and super-resolution imaging reveals enhanced cell surface clustering of HLA-I molecules

**Fig. 4.**
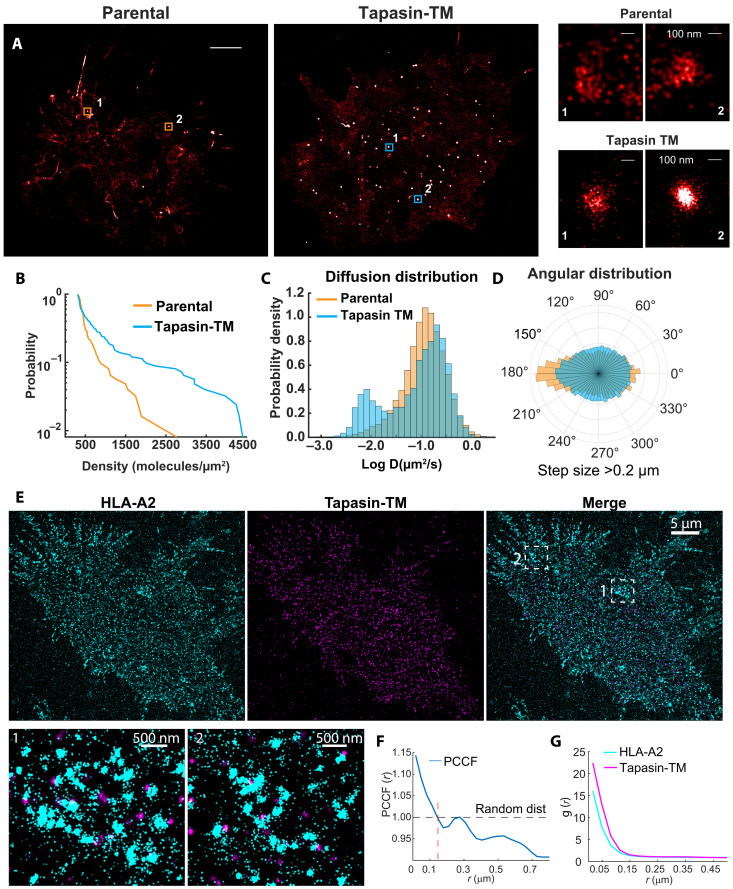
SMT and super-resolution imaging reveals tapasin-TM mediated clustering of HLA-I molecules. (**A**) Single molecule localization map of HLA-I molecules in Tapasin-TM (left), Parental (middle) with representative magnified views of (right) high density regions revealing differences in local density. Coloring represents degree of density with white indicating higher density. Scale bar, 5 µm. (**B**) Distribution of cluster density between tapasin-TM and parental reveals higher fraction of dense clusters of HLA in tapasin-TM compared to parental cells. (**C**) Distribution of diffusion coefficients from single molecule trajectories of HLA-I in tapasin-TM and parental cells. (**D**) Polar histogram plot of angles between consecutive jumps in tracks from mobile molecules showing a shift from biased directional jumps along a line in parental cells to a more isotropic distribution is in tapasin-TM cells. (**E**) Super-resolution DNA-PAINT images showing expression of tapasin-TM (right) and HLA-A2 (left). Insets show representative regions depicting spatial relationships between tapasin-TM and HLA-A2 molecules. (**F**) PCCF showing degree of colocalization and coclustering of HLA-A2 and tapasin-TM molecules. A PCCF of 1 indicates a random distribution, while values >1 indicate correlation and values <1 indicate exclusion. (**G**) Pair correlation function [g(*r*)] analysis showing clustering of HLA-A2 or tapasin-TM molecules at scales below 150 nm. Approximately 300,000 localizations were analyzed.

To further quantify changes in the organization of HLA-I molecules on the plasma membrane, we compared the nanoscopic distribution and mobilities of HLA-I molecules in tapasin-TM and parental lines using live-cell single-molecule tracking (SMT). Spatial mapping of single-molecule localizations shows that HLA-I molecules form both a higher number and density of HLA-I clusters in tapasin-TM cells, relative to parental cells (Fig. 4, A and B, and fig. S7). Quantification of single-molecule kinetics shows that HLA-A2 molecules from cells expressing tapasin-TM exhibited lower mobility, as measured by their diffusion coefficients (Fig. 4C and fig. S7, C and D). We also noted changes in the partitioning of HLA-A2 molecules between parental and tapasin-TM–expressing cells. HLA-I single-molecule tracks in parental cells tended to partition along linear protrusions on the membrane, which was reduced in tapasin-TM–expressing cells (Fig. 4D). To quantify this differential partitioning, we calculated the angle between consecutive jumps in tracks from mobile molecules. By comparing the ratio of angles in (0° ± 30°) and (180° ± 30°) to (90° ± 30°) and (270° ± 30°), we calculated a linearity metric that represents the fraction of tracks moving in a linear pattern. The linearity metric comparison revealed a higher fraction of HLA-I molecules in plasma membrane protrusions in parental cells (fig. S7E).

Next, we sought to establish the cell surface relationship between tapasin-TM and HLA-I molecules. To do this, we used two-color DNA-PAINT in fixed samples (*30*), which can enable super-resolution microscopy at the scale of tens of nanometers (*31*). Using this complementary method to our live-cell super-resolution microscopy, we found that tapasin-TM forms domains that partially cocluster with HLA-A2 (Fig. 4E). These data are evident in the paired cross-correlation function (PCCF), which describes the degree of colocalization of these molecules (Fig. 4F). Accordingly, the PCCF plot reveals colocalization at distances up to 100 nm followed by coclustering at distances up to 300 nm, aligning with the observed distributions from the reconstructed images. Notably, the observed PCCF values align with those previously reported for co-localized proteins (*32*). Of note, clusters of tapasin-TM appeared to be denser than HLA-A2 molecules (Fig. 4G). Together, these results reveal differential localization, distribution, clustering, and mobility of HLA-I molecules in the presence of tapasin-TM when compared to parental cells. Thus, these data suggest that tapasin-TM has multiple effects on HLA-I trafficking and dynamics at the cell surface, increasing the quantity of HLA-I present at the cell membrane as well as their half-life and spatial organization into high antigen density “hubs.”

### Surface HLA-I remodeling by tapasin-TM leads to enhanced tumor killing by T cells

**Fig. 5.**
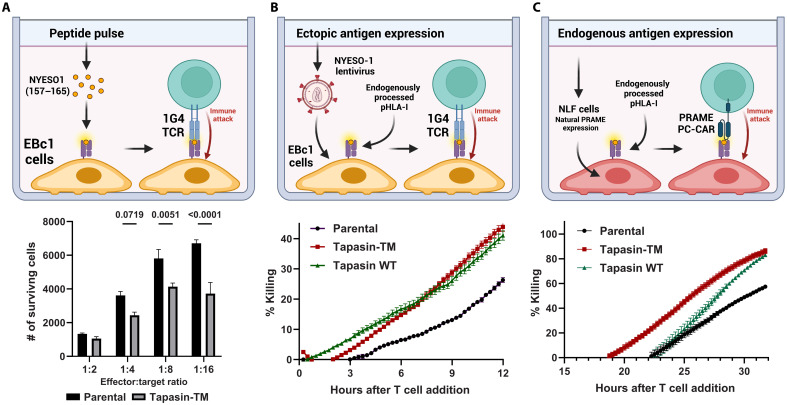
Tapasin-TM enhances killing of neuroblastoma cells by T cells targeting endogenous antigens. (**A**) Flow cytometry–based T cell killing assay. EBc1 cells were labeled with CellTrace Violet and were pulsed the following day with NYESO1_157–165_ heteroclitic peptide (C9V) (100 μg/ml) before addition of 1G4 T cells at different effector to target ratios (E:T). The number of surviving cells is then determined by addition of fluorescent counting beads and staining with a fixable live/dead viability dye (*N* = 3 technical replicates, error bars are SEM). (**B** and **C**) Impedance-based killing assays (*N* = 3 technical replicates, error bars are SEM). (B) EBc1 cells expressing full-length NYESO-1 with or without tapasin-TM were plated on gold electrode–coated tissue culture plates and 1G4 T cells were then added at a 1:2 E:T ratio and killing measured by loss of impedance. Percent killing was then determined by calculating the ratio of normalized cell index values with and without T cells. (C) NLF cells were plated as in (B), and HLA-A2/PRAME–specific PC-CAR T cells were then added at a 2:1 E:T ratio and killing monitored. Created in BioRender. Hwang, D. (2025) https://BioRender.com/f0iocky.

Tapasin-TM increased surface HLA-I expression and microdomain formation, which are both associated with enhanced T cell responses (*33*). We therefore tested whether EBc1 cells expressing tapasin-TM would be better recognized and killed by T cells. We first pulsed HLA-A2^+^ EBc1 cells with the NYESO1 heteroclitic peptide (NYESO1_157–165_;C165 to V) and incubated them with T cells expressing the NYESO1-specific HLA-A2 restricted T cell receptor (TCR) 1G4 (*34*). We observed that EBc1 cells expressing tapasin-TM were more readily killed than parental cells (Fig. 5A). To directly assess tapasin-TM effects on endogenously processed and presented antigens and their detection by T cells, we expressed the full-length NYESO-1 protein in adherent EBc1 cells and measured killing kinetics by 1G4 TCR T cells via change in cell culture plate impedance. Tapasin WT–transduced cells with similar HLA-I up-regulation levels were assessed in parallel experiments (fig. S8A). As expected, EBc1 cells expressing the antigen endogenously were killed more rapidly in tapasin-TM and tapasin WT–expressing cells, relative to the parental cells (Fig. 5B). We also tested the T cell killing kinetics of PRAME/HLA-A2^+^ NLF cells, which show higher up-regulation levels of HLA-I in tapasin-TM compared to WT tapasin–expressing cells (Fig. 2F). PC (peptide-centric) chimeric antigen receptor T (CAR T) cells specific for a PRAME/HLA-A2 antigen displayed enhanced killing of NLF cells expressing tapasin-TM when compared to both parental or tapasin-WT expressing cells (Fig. 5C), aligning with the observed levels of HLA-I up-regulation. Enhanced killing was not due to differences in growth curves or bystander killing (fig. S8. B and C). Thus, these data show that tapasin-TM effects on HLA-I molecules do not interfere with immune synapse formation and suggest an increase in the density of specific immunogenic peptide antigens on the cell surface.

### HLA-Shuttle enhances capture of the immunopeptidome in cold neuroblastoma cells

**Fig. 6.**
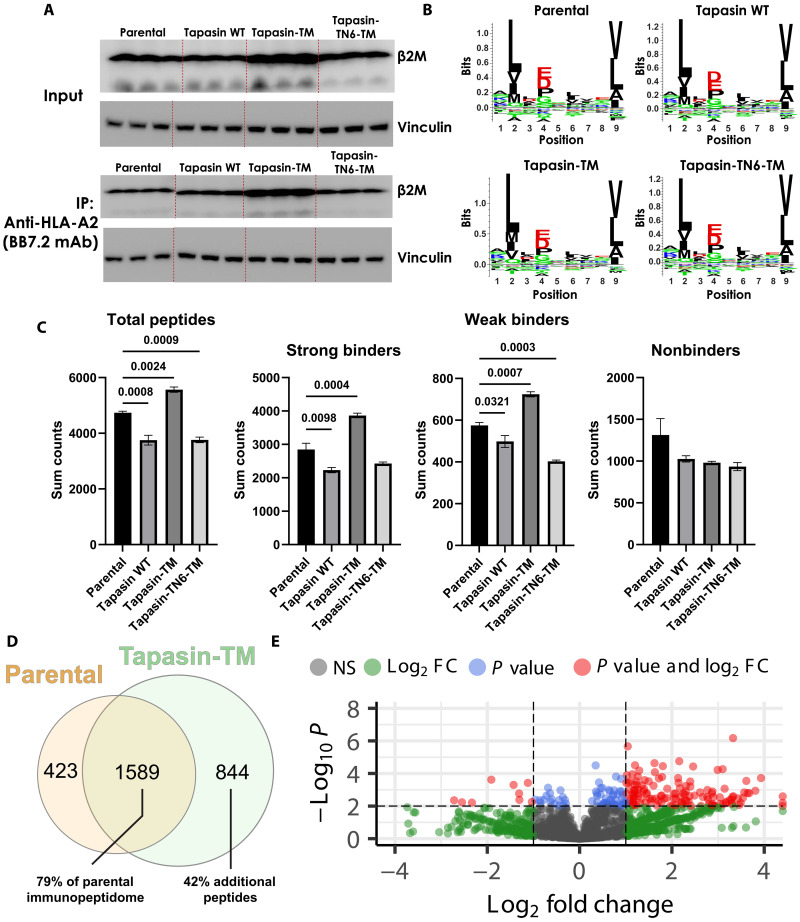
Tapasin-TM enhances capture of the immunopeptidome in a cold tumor setting. (**A**) IP of HLA-A2 with BB7.2 antibody from parental EBc1 or WT tapasin, tapasin-TM, or tapasin-TN6-TM–expressing cells. *N* = 3 replicates from cells harvested on different days and processed independently. Immunoblotting for light chain, β_2_m, to visualize quantity of HLA-I derived from those cells is shown. Vinculin was the loading control. Input lysates before IP and following IP with BB7.2 are shown. (**B**) Following peptide elution and timsTOF MS, sequence logos of all nine amino acid–long peptides were plotted with seq2logo. (**C**) Binding of 8 to 10 residue peptides to HLA-A2 was predicted by NetMHCpan4.1 (*36*). Data were then parsed into predicted strong, weak, and nonbinders. Quantification of sum counts of peptides detected in MS data where average of *N* = 3 replicates is shown. Counts represent instances during the MS run in which identifiable peptides were detected, a measure which is proportional to abundance. (**D**) Venn diagram showing distribution of unique 8- to 10-mer peptides isolated from parental EBc1 or tapasin-TM–expressing cells. Data represent pooled peptides from *N* = 3 replicates. (**E**) Volcano plots comparing change in abundance between parental and tapasin-TM–expressing cells. Log_2_ fold change in intensity (a measure which is proportional to abundance) versus −log_10_ of *P* values is depicted. Statistics were calculated using unpaired *t* test.

We hypothesized that the increased HLA-I stability conferred by tapasin-TM may enhance MS-based approaches to druggable target identification, which are often challenged by low signal to noise leading to a partial capture of the immunopeptidome (*10*). To test this, we immunoprecipitated HLA-A2 complexes using the conformation-specific anti–HLA-A2 antibody BB7.2 (*21*) from parental EBc1 cells and those expressing WT tapasin, tapasin-TM, and tapasin-TN6-TM. We selected WT and tapasin-TM–expressing cells with equivalent surface expression of HLA-I (fig. S8A), enabling us to perform a more direct comparison of the effects of tapasin-TM in enhancing HLA-I immunoprecipitation (IP). Following IP, we measured HLA-I by immunoblotting for β_2_m in triplicate, independently prepared samples, and found that tapasin-TM enhanced IP of HLA-A2 over WT tapasin (Fig. 6A). Eluted peptides were then identified using thermal ionization with time-of-flight MS (timsTOF), and analyzed using the MSFragger software (*35*). The length distributions of identified peptides showed that the expected preference for 9-mers (fig. S9), with peptide logos were characteristic of the HLA-A2–binding motif (Leu at position 2 and Leu/Val at position 9; Fig. 6B).

To further enrich for high-affinity binders, we focused all subsequent analysis on 8- to 10-mers, which contained the majority (>90%) of all identified peptides in the data (table S1). We parsed the data into strong, weak, and nonbinders using NetMHCpan4.1 (*36*). When comparing the total pool of peptides identified by MS between conditions, tapasin-TM had the highest enrichment of predicted binders (Fig. 6C). Using the list of individual peptides isolated from parental or tapasin-TM cells, we evaluated the degree of overlap and changes in intensity (a proxy for abundance) of shared peptides. Of the 2012 unique peptides identified in the parental Ebc1 cell line, 79% were also present in tapasin-TM–expressing cells, indicating robust capture of the native EBc1 immunopeptidome (Fig. 6D). Tapasin-TM expression also significantly increased the MS intensity of peptides common with parental EBc1 cells (Fig. 6E). Notably, we identified 844 additional peptides from tapasin-TM–expressing cells, which could not be detected in the parental line. These results support that tapasin-TM enhances capture of the immunopeptidome when introduced in cold tumor cells.

### Identification of therapeutically relevant tumor-associated antigens in neuroblastoma cells

**Fig. 7.**
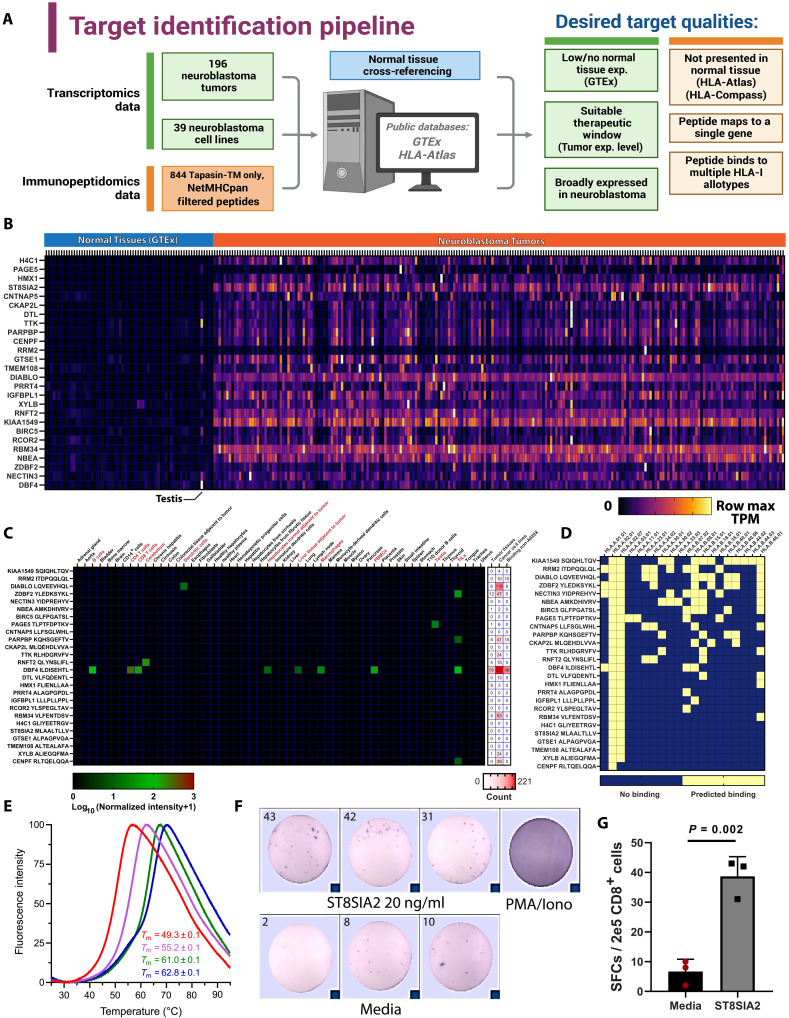
HLA-Shuttle reveals additional HLA-A2 neuroblastoma–associated antigens with favorable therapeutic windows. The 844 peptides identified exclusively in tapasin-TM expressing EBc1 cells were analyzed for clinically relevant targets. Data are *N* = 3 pooled replicates. (**A**) Multi-omics approach for target identification. Created in BioRender. Hwang, D. (2025) https://BioRender.com/23osb1t. (**B**) Heatmap of row max normalized TPM values from 68 normal tissues and 224 neuroblastoma tumors for the top 26 genes. Raw data for normal tissue obtained from GTEx portal and the Gabby Miller Kids First dataset. RRM2 is broadly expressed but is difficult to visualize because of a single high-expressing tumor. Testis and adrenal glands were included. (**C**) Mapping of candidate peptide presentation on healthy tissue using the HLA-Compass immunopeptidomic database (Alithea Bio). (left) Names of detected tissues are colored red. Log_10_ normalized MS signal intensity is shown. (right) Number of tumor tissue samples or cancer cell lines where each peptide was detected. Last column shows number of samples where peptide was detected from samples that did not carry HLA-A2 alleles. (**D**) Mapping of predicted binding of peptides to 20 common HLA-A*, B* allotypes using NetMHCpan 4.1 (*36*). (**E**) Thermal stability curves of HLA-A*02:01 refolded with the top identified tumor-associated peptides measured by differential scanning fluorimetry. Peptide identities and associated affinities predicted by NetMHCpan4.1 (*36*) are PAGE5 (red, 329 nM), CKAP2L (magenta, 118 nM), ST8SIA2 (green, 2.8 nM), and HMX1 (blue, 3.8 nM). Data are means ± SD for *n* = 3 technical replicates. (**F** and **G**) IFN-γ ELISPOT for human CD8 T cells cocultured with autologous peripheral blood mononuclear cell–derived dendritic cells pulsed with MLAALTLLV peptide. Cells stimulated with phorbol 12-myristate 13-acetate (PMA)/ionomycin was used as a positive control, and cells treated with media served as negative control. Number of spot forming cells (SPCs) per 200,000 cells is shown. Statistical significance was determined by unpaired *t* test.

Additional peptides identified using tapasin-TM may contain TAA targets that are of interest for immunotherapy development. To search for such antigens, we developed a multi-omics pipeline for identifying clinically relevant peptides by combining transcriptomic and immunopeptidome data from normal tissues, neuroblastoma tumors, and neuroblastoma cell lines (Fig. 7A). The 844 peptides unique to tapasin-TM–expressing cells mapped to 766 genes. We ranked these genes by low normal tissue expression using the GTEx database (*37*) and then eliminated genes with high expression in normal tissues. Next, we filtered out genes that were represented in the ligandomes of normal tissue using the HLA Ligand Atlas database (*38*). Last, we focused on genes showing overexpression in 196 neuroblastoma primary tumors (*39*) relative to healthy tissue, to eliminate targets with potentially insufficient therapeutic windows. From the resulting list of 26 remaining genes (Fig. 7B), we noted the presence of known, therapeutically relevant TAAs derived from PAGE5 (*40*), and HMX1 (*8*). We also performed a complementary analysis by referencing the genes mapped from our immunopeptidomics data using gene expression data from 39 common neuroblastoma cell lines (*41*), with consistent results. In total, 17 of 21 genes identified from referencing to the cell line data were also among the 26 genes identified from our origingal referencing to primary tumor data, further supporting the validity of the identified peptide targets (fig. S10A). As a final risk mitigation step, we used HLA-Compass (Alithea Bio), an immunopeptidomics database of healthy, tumor-adjacent benign, and cancer tissue/cell lines. Nearly all 26 candidate peptides were undetectable in healthy tissues (Fig. 7C). In most cases where peptides were found, they were detected in tumor-adjacent benign tissue, or TILs, which may not adequately represent noncancerous tissue. We found that 13 of 26 peptides were presented in other tumor tissue or cancer cell lines, including from samples that did not carry HLA-A2 (Fig. 7C), further suggesting that candidates may be presented by multiple HLA-I allotypes. Next, we sought to determine whether candidate peptides could potentially bind to other common HLA-I allotypes using NetMHCpan 4.1 (*36*) and found several peptides that could be presented by additional HLAs, beyond the A02 supertype (Fig. 7D). Last, to experimentally validate peptide binding on HLA-A2, we refolded recombinant heavy-chain HLA-A*02:01/β_2_m complexes with the PAGE5, CKA2PL, HMX1, and ST8SIA2-derived synthetic peptides and measured their protein thermal stability using differential scanning fluorimetry. For three of four peptides, we measured *T*_m_ values consistent with highly stable, properly conformed pHLA-I protein complexes (Fig. 7E) (*42*). Our analysis highlights potential immunotherapy targets including *ST8SIA2*, a developmental gene that participates in the biosynthesis of the established cancer-associated disialoganglioside GD2 (fig. S10B) (*43*). Similarly, a peptide expressed from *HMX1* forms a highly stable complex with HLA-A2 and has widespread expression across neuroblastoma tumors, in agreement with a previous study by our group (*8*).

To assess the immunogenicity of the ST8SIA2 (MLAALTLLV) and HMX1 (FLIENLLAA) peptides, we panned for CTLs specific for these peptides using peripheral blood mononuclear cells from HLA-A*02:01^+^ healthy donors. We were able to identify the presence of CD8 T cells secreting interferon-γ (IFN-γ) when cocultured with MLAALTLLV peptide–pulsed, autologous dendritic cells (Fig. 7, F and G). Similarly, preliminary experiments indicated that FLIENLLAA peptide–specific, IFN-γ–secreting T cells could also be detected (fig. S10C). These data further suggest that MLAALTLLV and FLIENLLAA are immunogenic TAAs presented by HLA-A2. Together, our data suggest that dysregulation of the HLA-I pathway in cold tumors challenges the identification of a considerable fraction of the immunopeptidome, including TAAs, which may be therapeutically relevant. Thus, HLA-Shuttle enables recovery of additional TAAs in cold tumor cells.

### HLA-Shuttle enhances tumor-associated antigen discovery across HLA allotypes

**Fig. 8.**
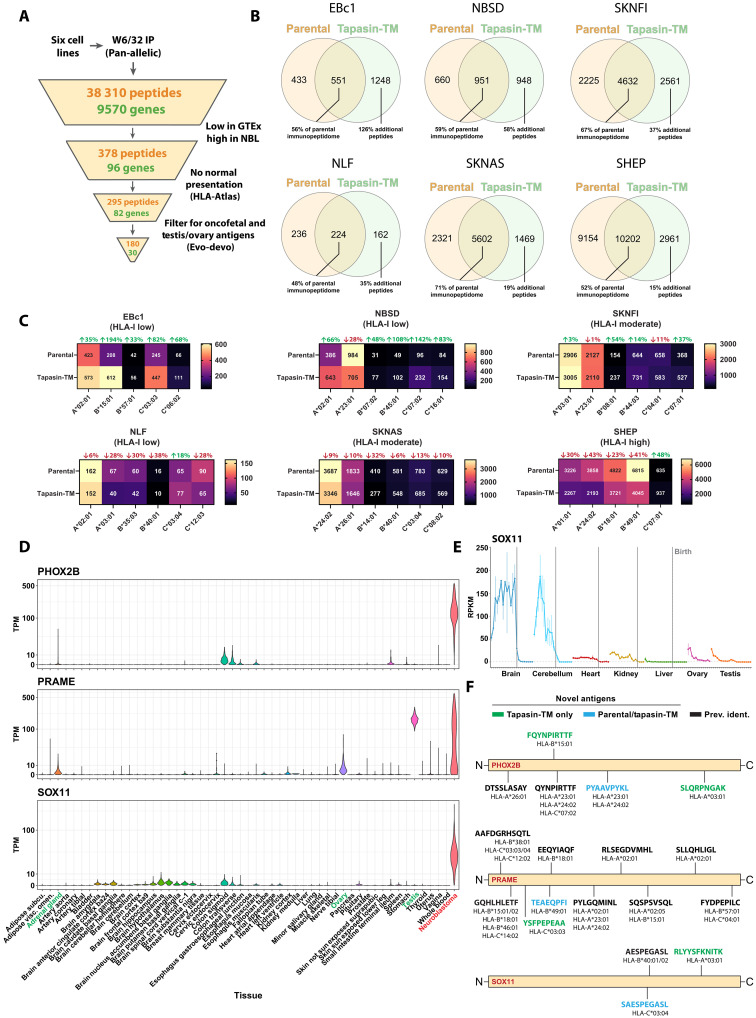
Tapasin-TM shows multipronged effects on neuroblastoma immunopeptidomes and enables discovery of potential targets across HLAs. HLA-I complexes from parental and tapasin-TM–expressing neuroblastoma cell lines were immunoprecipitated using W6/32 antibody, and peptides were eluted for timsTOF MS. (**A**) Bioinformatics pipeline and filtering of data. (**B**) Venn diagrams depicting overlap between parental and tapasin-TM–expressing cell lines for 8- to 11-mers that were used for analysis. (**C**) Peptides were computationally assigned to HLA-I alleles from the genotype of each cell line. Peptide counts stratified by HLA-I allele are shown. Changes in percentage of peptides relative to parental counts are shown in red or green for percent increases and decreases respectively. (**D**) Our bioinformatics pipeline was initially applied to combined peptide data (38,310 peptides) as in Fig. 7A. An additional filtering step was implemented using the Evo-devo (*50*) developmental gene expression database to remove genes with expression after birth. In place of HLA-Compass, the PCI-DB database (*52*) was used to filter out peptides presented on normal tissue. Gene expression plots in normal and primary neuroblastoma tumors for cancer immunotherapy targets PHOX2B, PRAME, and target SOX11. (**E**) Expression of SOX11 in reads per kilobase of transcript per million mapped reads (RPKM) across lifespan. Gray dotted lines indicate birth. (**F**) Mapping of potentially therapeutically relevant tumor-associated antigens isolated from our immunopeptidomics data for PHOX2B, PRAME, and SOX11. The HLA-I restriction of these peptides based on experimental data derived from IEDB (*51*), PCI-DB (*52*), and this study are listed. Peptides are also colored on whether these epitopes were previously known to be presented in tumor tissue (via IEDB and PCI-DB), and previously unknown antigens. Peptides isolated from parental or parental and tapasin-TM cells are colored blue. Previously unknown peptides only isolated from tapasin-TM–expressing cells are colored green.

Our IP of HLA-A2 complexes using the BB7.2 antibody was useful for the initial validation of HLA-Shuttle as a tool for enhancing immunopeptidome capture, focusing on a clinically relevant HLA allotype. However, most antigen discovery campaigns use the W6/32 HLA-I pan-allelic antibody, followed by computational prediction of antigen binding to the HLA-I alleles present in the genotype of the analyzed sample (*44*). Moreover, several questions remain about the utility of HLA-Shuttle regarding its HLA-I–producing versus –stabilizing effects and how this would influence antigen capture across cells with different HLA genotypes and baseline expression levels. Thus, to provide clarity to these application relevant questions, and to potentially uncover neuroblastoma antigens, we performed a W6/32-based immunopeptidomics campaign on six of the seven neuroblastoma cell lines we evaluated initially, which span baseline HLA-I levels and tapasin-TM–mediated effects on HLA-I expression, and stability (Figs. 2F and 3). Among these cell lines was the HLA-I high cell line SHEP, which is derived from a mesenchymal neuroblastoma tumor, a rare subtype with high immunogenicity and high expression of APP proteins, including tapasin (*45*).The immunopeptidomes from both parental and tapasin-TM–expressing cell lines were captured and used in subsequent analysis (table S2). In total, 38,310 8- to 11-mer peptides were identified (Fig. 8A and fig. S11), with stark differences in the number of peptides identified per cell line, which correlated closely with their corresponding baseline HLA-I levels (Figs. 8B and 2F). Capture of the immunopeptidome in tapasin-TM–expressing cells relative to the corresponding parental cells was increased by an average of 59% (ranging from 48 to 71% in the six cell lines analyzed). Despite using HLA-Shuttle to boost surface HLA-I levels, capture of the immunopeptidome of the HLA-I low cell line NLF was limited to several hundred peptides, as opposed to thousands from other HLA-I low cell lines (EBc1 and NBSD). In line with previous reports (*46*), capture of the immunopeptidome with W6/32 was less efficient than with BB7.2, as evidenced by 2232 total peptides restricted to five HLA alleles in EBc1 cells captured by W6/32 IP (Fig. 8B) compared to 2856 HLA-A2–restricted peptides captured from the same cell line using BB7.2 (Fig. 7D). However, tapasin-TM–expressing EBc1 cells maintained a pronounced expansion of the immunopeptidome by 126%, relative to parental cells, which was consistent across five HLA allotypes (Fig. 8C). In NBSD and NLF cells, we isolated 58 and 35% more peptides when using HLA-Shuttle (Fig. 8B). Strikingly, the HLA-I moderate line SKNFI, which did not up-regulate HLA-I production but did display enhanced HLA-I cell-surface stability when transduced with tapasin-TM, showed a 35% increase, suggesting a more qualitative effect in the immunopeptidome. Last, for the HLA-I high cell line SHEP (*45*), we observed a contraction of the immunopeptidome, likely due to the known peptide repertoire focusing effects of tapasin (*26*), which are amplified by tapasin-TM overexpression in cells with robust antigen presentation. These results highlight multipronged effects of using HLA-Shuttle in different cellular states and HLA backgrounds, which can be leveraged to enhance antigen discovery campaigns.

Next, we further stratified our results using a computational assignment of peptides to putative HLA-I alleles present in the genotype of each cell line (Fig. 8C). These data reveal notable patterns in allele-specific upregulation of peptides. In HLA-low cells EBc1 and NBSD, the number of identified peptides increased in 10 of the 11 alleles covered. HLA-C*–restricted peptides appeared to benefit the most from tapasin-TM expression, with five of six cell lines showing robust increase for at least one HLA-C* allele. Demonstratively, this was also consistently observed for the SHEP cell line, where the only allele with increased peptide numbers in tapasin-TM–expressing cells was HLA-C*07:01, showing a 48% increase. Peptides restricted by HLA-C*07:01/02 alleles were increased in all three cases, in HLA-I low NBSDs, HLA-I moderate SKNFIs, and HLA-I high SHEP cells. Given that HLA-C* expression is lower relative to HLA-A*, B* alleles and varies widely among individuals (*47*), this result further supports the potential of applying HLA-Shuttle in antigen discover campaigns for personalized vaccine and immunotherapy development.

Last, we sought to determine whether HLA-Shuttle could be used to enhance identification of therapeutically relevant antigens across HLAs using our in-house bioinformatics pipeline (Fig. 7A). Given the multipronged effects of tapasin-TM observed in the immunopeptidomes of the six neuroblastoma cell lines (Fig. 8, B and C), we chose to analyze the full set of 38,310 pooled peptides identified in both parental and tapasin-TM–expressing cells (Fig. 8, A, D to F). Upon mapping the list of peptides to genes and referencing those genes for tissue expression levels, we observed several known cancer targets with favorable therapeutic windows, including PHOX2B (*8*), PRAME (*48*), and SOX11, a primarily developmental transcription factor expressed in many different tumors (Fig. 8D) (*49*). We also confirmed these targets as primarily onco-fetal or as cancer testis antigens by the Evo-devo database (*50*), which offers transcriptome data for key organs during fetal development and across lifespan (Fig. 8E). We then searched our top peptide candidates against the IEDB (*51*) and PCI-DB (*52*) immunopeptidomics databases to further select for targets that are present in the immunopeptidomes of cancerous but not in relevant normal tissues. Among the top selected peptides, we identified three previously unknown antigens derived from *PHOX2B* expression, two of which could only be identified in tapasin-TM–expressing cells. Similarly, previously unknown antigens were uncovered for the PRAME and for SOX11 gene products, several of which were only captured in tapasin-TM cells (Fig. 8F). Together, these data suggest that HLA-Shuttle may be used as an APP pathway enhancer, to reveal additional public antigens spanning multiple clinically relevant HLA allotypes.

## DISCUSSION

HLA-Shuttle is a system for enhancing antigen presentation in cells with severe APP pathway deficits through multimodal chaperoning or shuttling effects that ultimately result in greater steady-state quantities of stable pHLA-I persisting on the cell surface. HLA-Shuttle exploits the widespread down-regulation of tapasin in cancers, both restoring tapasin expression in these cells while additionally stabilizing HLA-I molecules through its extra-ER activity, thereby providing a continuum of chaperoning activity across the secretory pathway. To benchmark its utility, we tested our method in neuroblastoma cells, which represent exceptionally nonimmunogenic cancers that have been difficult to target with immunotherapy, both due to low natural immune response against the cancer, but also due to the paucity of known targetable antigens (*53*, *54*). Nevertheless, neuroblastoma tumors are likely to be highly responsive to adoptive cell immunotherapies, as evidenced by recently completed clinical trials targeting GD2 with CAR T cells, which showed high efficacy in maintaining long-term disease-free remission, despite the use of first-generation CARs lacking costimulatory domains (*55*). Intrinsically noninflamed cancers are hypothesized to not undergo extensive immunoediting (*56*) and may therefore be highly susceptible to T cell killing if detected. Thus, for this class of tumors, the development of tools and systems that boost antigen presentation may enable greater immunological recognition by the immune system while also enhancing targetable antigen discovery campaigns for adoptive cell therapy. We show that HLA-Shuttle may address this issue by bolstering T cell killing and by enabling identification of potentially relevant TAAs that were undetectable in the unmodified cells.

We identify two probable mechanisms by which HLA-Shuttle boosts production of stable pHLA-I. First, tapasin appears to act in its native capacity as a key component of the PLC, albeit likely without binding to TAP proteins, as its association with TAP is known to rely on interaction with K452, which is absent in tapasin-TM (*57*). This is not without precedent, as soluble tapasin has been shown to largely rescue tapasin-mediated HLA-I production (*58*). Second, our trafficking and stability experiments revealed that tapasin-TM had a direct stabilizing effect on cell-surface HLA-I molecules that could not be recapitulated with expression of WT tapasin. We attribute this to trafficking of tapasin outside the ER, suggesting a model whereby tapasin reprises its ER-native role of stabilizing empty or suboptimally loaded HLA-I molecules (*59*), but in other cellular compartments of the secretory pathway. Intriguingly, our single-particle tracking of HLA-A2 molecules revealed not only a higher abundance of HLA-A2 molecules on the surface of the tapasin-TM–transduced cells but also their reorganization into microdomains that are known to orchestrate efficient immunosurveillance and T cell activation (*33*). It is unknown whether tapasin-TM is actively participating in this change at the cell surface or whether this is a consequence of enhanced overall production of a more stable HLA-I. However, our data showing that tapasin-TM colocalizes with HLA-A2 in partially overlapping clusters lend credence to the former hypothesis. These data suggest that tapasin-TM may act as a dynamic sensor of the folding state of HLA-I, facilitating the refolding of HLA-I directly on the cell surface and enhancing its stability, as it does in the ER (*60*). Further experiments are needed to fully delineate the mechanism underlying the enhanced stability of HLA-I at the plasma membrane in cells expressing tapasin-TM.

As expected, the efficacy of tapasin-TM in facilitating production and stabilization of specific HLA-I alleles largely align with previous reports, showing large effects on highly tapasin-dependent allele HLA-A*01:01 and modest effects on tapasin-independent allele HLA-A*68:01 (*26*). Some notable exceptions were HLA-A*02:01 and HLA-C*07:02, although conflicting reports do exist showing that HLA-A2 molecules rely on tapasin for efficient production and presentation (*60*). Last, the HLA-I up-regulation effects of tapasin-TM were also dependent on ERp57, a ER protein that stabilizes tapasin and, together with tapasin, acts to efficiently recruit HLA molecules and facilitate peptide binding and repertoire optimization (*61*). These results suggest that tapasin-TM–mediated up-regulation of HLA-I may be rate-limited by the levels of ERp57 available in neuroblastoma cells. If proven true, even greater up-regulation and HLA-I stabilization may be possible by coexpression of cochaperones ERp57 and/or calreticulin, which together with tapasin form the core of the PLC (*62*). These data also highlight that in its current incarnation, HLA-Shuttle is unlikely to be efficacious for certain APP defects. For example, loss of β_2_m, HLA-I loss of heterozygosity, and other critical APP machinery known to have detrimental effects on the immunopeptidome (*63*) are also likely to circumvent the effects of tapasin-TM. To address those issues, complementary technologies would need to be developed and rigorously tested in future studies of the immunopeptidome.

Tapasin-TM expression enhanced the immunogenicity of EBc1 and NLF cells, leading to earlier engagement and killing by T cells. Given that initial engagement of neuroblastoma tumors often never occurs because of exceptionally poor immunosurveillance, we suspect that faster engagement is indicative of more robust immunosurveillance, which is desirable for treating neuroblastoma. However, we also observed relatively similar rates of killing between parental and tapasin-TM–expressing cells after the initial engagement. These data were somewhat discordant with direct effects on HLA-I production and stability, which were seemingly more robust. However, the overall quantity of antigen presentation in these cells is still low by comparison to hot tumors. Thus, the difference in T cell signaling and activation induced by HLA-Shuttle–expressing cells is likely to be comparatively weaker than its effects on the stability of HLA-I molecules that are being produced. Nevertheless, the higher HLA-I density on cells expressing HLA-Shuttle can lead to faster engagement and killing of the tumors. A relevant example of the importance of target engagement in cold tumors is the high susceptibility of neuroblastomas to T cell–mediated killing [as evidenced by the great success of anti-GD2–directed CAR T cells (*55*)], contrasted with the abysmal efficacy of immune checkpoint inhibitors (*64*). These data may imply that treatments that increase HLA-I production in cold tumors, potentially including HLA-Shuttle if sufficiently developed in future work, can be disproportionately efficacious in vivo by comparison to in vitro. In line with this concept was a human clinical trial where IFN-γ treatment restored T cell infiltration into the tumors, indicating that up-regulated HLA-I was the cause of substantially improved T cell trafficking to the tumor site (*65*). As a relevant note for potential in vivo applications, HLA-Shuttle also had little effect on total surface HLA-I levels in cells with higher, presumably more normal HLA-I production. This could suggest that application of this technology in vivo may have relatively benign off-target effects. If proven true, these features may mitigate any potential risk of eliciting autoimmunity, especially if combined with targeted delivery strategies such as lipid nanoparticles, which leverage established cancer surface markers, including GD2 (*66*).

HLA-Shuttle enhanced capture of the HLA-A2 immunopeptidome from EBc1 cells. Greater quantities of HLA-A2 complexes could be pulled down from cells expressing tapasin-TM by comparison to cells expressing WT tapasin, despite similar surface levels of HLA-A2 by flow cytometry. These data further imply that tapasin-TM acts to globally stabilize the folded conformation of HLA-I, prolonging its lifetime on the cell surface. At the peptide level, tapasin-TM facilitated capture of the greatest number of peptides with overall higher abundance per peptide. These data also align with previous work showing that knockout of key APP machinery, including tapasin, influences the observable immunopeptidome (*63*). These data may suggest that tapasin-TM facilitates peptide loading in the ER but also stabilizes low-affinity or low-abundance peptide–HLA-I complexes sufficiently to enable their identification by MS. This model aligns well with the notable increase in HLA-A2 peptides captured in tapasin-TM–expressing cells, leading to a 42% expansion of the observable HLA-A2 immunopeptidome. This approach offers advantages over other methods of increasing HLA-I such as IFN treatment, which induces hundreds of genes across many cellular pathways (*67*), changing the protein expression profiles of those cells, while also fundamentally altering proteasomal production of peptides through induction of the immunoproteasome (*68*). These effects combined lead to a more complex immunopeptidomic landscape, challenging the identification of relevant antigens, which may also presented in the parental cells under native conditions. In contrast, HLA-Shuttle leverages a more targeted approach, where any induced effects in the immunopeptidome are largely limited to peptides that are already processed and trafficked through the cell’s endogenous pathway.

Expression of tapasin-TM enabled identification of 844 additional HLA-A2–restricted peptide antigens not detectable in the parental cell line. Following filtering for absent expression/presentation in normal tissues, and overexpression in neuroblastoma, we arrived at 24 candidate peptides. Among this list were two established TAA’s with ongoing therapeutic interest, a peptide derived from cancer testis antigen PAGE5 (*40*) and HMX1 (*8*). Both *PAGE5* and *HMX1* are developmentally/germ line–linked genes, with low or absent expression in normal adult tissues. Our analysis also revealed a candidate peptide from *ST8SIA2*, a gene overexpressed across cancers that participates in tumor invasiveness (*69*). Like *PAGE5* and *HMX1*, *ST8SIA2* is primarily expressed during fetal development, without notable expression in normal tissues (*69*). Another notable example was *CKAP2L*, which clusters together with several genes identified from referencing of our immunopeptidomics data, including *BIRC5*, *BRCA2*, and *CENPF*, forming a cluster of genes associated with cell proliferation across cancers (*70*). Moreover, many of the peptides we identified were found in the HLA-Compass database as being presented by tumor tissues and cell lines, indicating that these are genuinely presented peptides that fall below the threshold of identification by typical immunopeptidomics workflows. The peptides derived from PAGE5, HMX1, ST8SIA2, and CKAP2L could be refolded with HLA-A2, albeit with a range of stabilities, confirming their ability to bind. Validation with isotope-labeled peptides will be needed to confirm their presence in these cells. Last, our early TCR discovery efforts indicate that the peptide derived from ST8SIA2 is suitably immunogenic, suggesting the absence of strong tolerogenic mechanisms, and aligning with larger literature on cancer-specific expression of ST8SIA2, and our own bioinformatics analysis on its presentation in cancers and normal tissues.

Our pilot study using tapasin-TM as an enhancer for immunopeptidomics-based target discovery had several limitations, notably including the use of folded HLA-A2–specific antibody BB7.2. We made this choice to simplify our initial analysis by focusing on a single HLA allele and because of the higher effectiveness in HLA-I IP with BB7.2 than with the pan–HLA-I antibody W6/32 (*46*). Nevertheless, its use also excludes peptides presented via other HLA alleles. To further explore the effects of HLA-Shuttle on enhancing peptide capture across a range of alleles, baseline HLA-I levels, and tapasin-TM–mediated effects, we immunoprecipitated total HLA-I complexes from six neuroblastoma lines using W6/32. These data reveal patterns indicating that HLA-Shuttle efficacy is controlled by a combination of allele composition and baseline HLA-I (which reports on the overall functionality of the APP pathway). In two of the three HLA-low cells, tapasin-TM elicited strong increases in the number of peptides derived from 10 of 11 HLA-I alleles. However, baseline HLA-I levels did not appear to be the only driving factor for increases in the peptide repertoire. When stratified on a per-allele basis, substantial increases in the immunopeptidomes eluted from the highly tapasin-dependent HLA-B* alleles HLA-B*44:03 and HLA-B*08:01 (*26*) were observed even in the HLA-I moderate cell line SKNFI, which did not up-regulate HLA-I when expressing tapasin-TM. Notably, SKNFI cells showed tapasin-TM mediated increases in HLA-I stability, suggesting a tapasin-mediated stabilization of the folded conformation for these B* alleles. Moreover, we noted widespread increase in peptide numbers from HLA-C* alleles, potentially due to their notably lower binding affinity for their peptides (*71*), necessitating chaperoning activity from tapasin to enable sufficient survival of these complexes to be identified via timsTOF MS. This was particularly noteworthy for the HLA-C*07:01/02 alleles, where peptide numbers for it increased in all instances, across baseline HLA-I levels, with robust increases observed even in the SHEP HLA-I high, tapasin high line. Last, we observed repertoire contraction in SHEP cells, which we posit is due to repertoire focusing effects of tapasin (*26*, *72*). The degree of repertoire focusing we observe in SHEP cells is likely due to the already high endogenous expression of tapasin in SHEP cells (*45*), along with overexpression of additional tapasin via HLA-Shuttle, which amplifies this function of tapasin. Together, these data imply a multipronged mechanism for the effect of tapasin-TM on cells: (i) In cell lines with a dysfunctional APP and low parental HLA-I expression, tapasin-TM results in increasing the total number of ligands by partially restoring a functional APP and by creating a continuum of chaperoning activity along the secretory pathway. (ii) In cell lines with normal/high parental HLA levels, tapasin-TM “focuses” the immunopeptidome, likely toward higher-affinity peptides (*59*), leading to an apparent decrease in the total number of identifiable peptides. (iii) Both of the above mechanisms are subject to the HLA-I haplotype of the cells in question, owing to the well-established HLA allelic dependence to tapasin (*26*). Thus, in some scenarios, such as highly tapasin-dependent HLA-B* and potentially C* alleles, mechanism (i) may disproportionately contribute over mechanism (ii), thereby resulting in the allele-specific benefits we observed in HLA-I normal/high cells.

Last, our results demonstrate the influence of HLA-I production and stability on immunopeptidome capture, with an immense range of peptide numbers isolated from HLA-I low to HLA-I high cells (from <1000 to ~20,000 peptides). These data further underscore the necessity for methods of enhancing presentation of these peptides in cold tumors, as it is likely that numerous therapy-relevant antigens remain below the limit of detection using current approaches. In conclusion, we herein present a technology for enhancing the capture and identification of peptide–HLA-I complexes in a targeted manner, by leveraging engineered components of the APP pathway. Our work provides a toehold for the identification and targeting of disease-associated antigens and neoantigens in settings where deregulated HLA-I APP is a hallmark of immune evasion, including cancer and latent infections.

## MATERIALS AND METHODS

Complete descriptions of all experimental methods and materials are available in the Supplementary Materials.

### Lentiviral production and transduction

Lentivirus was produced by cotransfection of Lenti-X 293T cells (Takara Bio) with pSFFV transfer vector containing gene of interest along with psPAX2 packaging vector and pMD2.G envelope vector. Transductions were performed by adding concentrated lentivirus to cells, and transduction efficiency was determined by intracellular staining using a phycoerythrin (PE)–conjugated anti-DYKDDDDK (FLAG) Tag Antibody (L5, BioLegend).

### CRISPR-Cas9 editing

The 721.221 cells expressing a single HLA-I allele were edited by electroporation of CRISPR-Cas9 ribonucleoproteins (RNPs). Cas9 was complexed with *TAPBP*-specific single guide RNA (sgRNA) 5′-GAACCAACACUCGAUCACCG-3′ (Synthego/Editco) at a 1:3 ratio Cas9/sgRNA and electroporated using a Lonza 4D-Nucleofector X Unit program EH-100 and buffers from p3 primary cell 4d-nucleofector x kit (Lonza). RNPs were prepared by adding in the following order: 5 μl of P3 buffer, 1.2 μl of sgRNA (100 μM), and 2 μl of Cas9 (20 μM) per reaction. An amount of 8.2 μl of this mixture was added to 200,000 cells resuspended in 15 μl of P3 buffer. Reactions were performed in 16-well strips, taking care that the liquid fully cover the bottom of wells and that no bubbles were introduced.

### HLA-I trafficking and internalization assays

EBc1 cells engineered with or without cyan fluorescent protein constructs were plated in complete RPMI 1640 and treated with either cycloheximide (Millipore Sigma #66-81-9), epoxomicin (Millipore Sigma, #324801), bafilomicin A1 (Millipore Sigma, #SML1661), brefeldin A (BioLegend, #420601), or vehicle. Cells were harvested and analyzed by flow cytometry. For internalization assays, the cells were stained with anti–HLA-A2 antibody and kept at 4°C to suspend HLA internalization. At respective time points, the cells were transferred to 37°C media to reinitiate HLA internalization process. The cells were then stained with LIVE/DEAD Fixable Near-IR stain (Invitrogen, #L10119) and then fixed. A secondary antibody stain (PE goat anti-mouse immunoglobulin G, BioLegend, #405307; allophycocyanin anti-Flag, BioLegend, #637307) was performed before flow cytometry acquisition.

### Super-resolution microscopy and SMT

EBc1 cells were plated on 35-mm glass bottom dish (Cellvis) and stained with Alexa Fluor 647–conjugated anti–HLA-A2 antibody (BB7.2, BioLegend) in 1 ml of Dulbecco’s phosphate-buffered saline (DPBS)+ calcium chloride + magnesium chloride (Gibco) and 2% bovine serum albumin (Miltenyi Biotec) for 25 min. Before imaging, cells were washed and submerged in dSTORM buffer, a phosphate-buffered saline buffer containing 10% w/v dextrose, glucose oxidase (500 μg/ml, Millipore Sigma), glucose catalase (40 μg/ml, Millipore Sigma), and 35 μl of 1 M 2-Mercaptoethylamine (MEA). Single-molecule imaging experiments were performed on a custom HILO microscope built on an Olympus TI-83 inverted microscope using a total internal reflection fluorescence objective (UplanApo, Oil HR). Single-molecule localization microscopy analysis was performed using INSIGHT software (Zhuang Laboratory, Harvard). Super-resolution images (Fig. 4E) were generated two-dimensional (2D) Gaussain corresponding to the detected localization of molecules using INSIGHT software. SMT was performed using trackit (*73*). For diffusion mapping the mean squared displacement of each track for each lag time was computed and fitted with 2D random diffusion model to extract diffusion coefficients (*D*), and the diffusion distribution was validated using state array–based single-particle tracking (*74*). PCCF and g(*r*) calculation were done using custom-written MATLAB script as described in (*32*). The PCCF and g(*r*) is a mean of 20 randomly selected circular regions of interest with 5-μm radius within cell boundary.

### Isolation of HLA ligands, analysis by LC-MS/MS, and raw data processing

HLA-I molecules were isolated using standard immunoaffinity purification methods as previously described (*8*) with minor mortification. Liquid chromatography–MS (LC-MS) data were acquired on a nanoElute 2 system connected to a timsTOF Ultra 2 using data-dependent Parallel Accumulation and Serial Fragmentation mode. Peptides were separated on an IonOpticks 25 cm by 75 μm Aurora Elite C18 column, maintained at 50°C. The tandem MS (MS/MS) raw files underwent processing using MsFragger v.22.0 with MSBooster (*35*). For this analysis, we used a reference of the human proteome from UniProt, consisting of Swiss-Prot and TrEMBL entries, supplemented with a list of 245 common protein contaminants. MS1 quantification was performed in IonQuant (*75*) without conducting a match between runs.

### Multiomics analysis of identified peptides

Transcriptome data (RNA sequencing) from 224 patient-derived neuroblastoma tumors and 39 human neuroblastoma cell lines were used along with immunopeptidomic data derived from the 844 peptides found in tapasin-TM cells but not parental cells. Genes associated with peptides were then used to rank data based on normal tissues expression using the GTEx version 10 database. Ranking was performed using rank() in base R programming language. The top 100 genes with the lowest normal expression were then further filtered to remove genes with low median transcript expression in neuroblastoma (average TPM <5 and by removing genes with TPM >20 in any single normal tissue excluding testis and adrenal gland. Testis was excluded as it is a source of developmentally related antigens, and adrenal gland was excluded as adrenalectomy is a common neuroblastoma treatment. Genes were then filtered at the peptide level by removal of those presented in normal tissue using the HLA-Atlas database. Last, peptides mapping to multiple genes were removed and peptides further analyzed with NetMHCpan 4.1 for binding to 20 common HLA-I alleles and presentation in normal and cancerous tissues using HLA-Compass (Alithea Bio).

A similar bioinformatics pipeline was implemented for the immunopeptidomics campaign using W6/32 antibody for HLA-I complex IP. Following filtering on low normal expression and high neuroblastoma tumor expression, and removal of genes associated with peptides presented in normal tissue excluding testis, thymus, ovary, and adrenal gland via cross-referencing with HLA-Atlas, we performed an additional filtering step to remove genes with notable expression after birth using the Evo-devo mammalian organ expression database (*50*). Last, peptides mapping to multiple genes were removed.

### Ethical approval and informed consent

The Human Immunology Core at the University of Pennsylvania provided all primary cells used in this study. The studies involving human participants were reviewed and approved by the University of Pennsylvania review board. A written informed consent to participate in this study was provided by the participants.

## Supplementary Materials

### This PDF file includes:

Supplementary Text Figs. S1 to S11 References
