# Supplementary material for: HLA-Shuttle: A system for enhancing antigen presentation in immunologically cold tumors
Source: Sci Adv. 2026 Jan 1;12(1):eaeb0821. doi: 10.1126/sciadv.aeb0821 (PMC12757076; doi:10.1126/sciadv.aeb0821)
Supplement: Supplementary file 1 — Supplementary Text Figs. S1 to S11 References [file sciadv.aeb0821_sm.pdf]

Science Advances
AAAS

Supplementary Materials for

HLA-Shuttle: A system for enhancing antigen presentation in immunologically cold tumors

Daniel Hwang *et al.*

Corresponding author: Nikolaos G. Sgourakis, nikolaos.sgourakis@pennmedicine.upenn.edu

Sci. Adv. **12**, eaeb0821 (2026)
DOI: 10.1126/sciadv.aeb0821

This PDF file includes:

Supplementary Text
Figs. S1 to S11
References

Extended Methods and Materials

Bioinformatics analysis of normal and cancerous tissue

Heatmap was generated using TPM expression data sourced from gene_tpm_2017-06-05_v8 Tissue expression data from The Broad Institute's Genotype-Tissue Expression (GTex) Data portal. Tumor TPM Expression levels were sourced from DepMap 24Q4 Omics Expression Batch Corrected Data from Primary tumor cell lines. Aggregate expression data in tumor cell lines was associated with aggregate expression data from healthy cell lines of the same tissue of origin. Percent change was calculated and plotted on a heatmap using seaborn in Python 3.14.

Cell Lines

SHEP, SKNAS, EBc1, NBSD, NLF, SKNFI, and SKNBE2C cell lines were obtained as a gift from the laboratory of John Maris.

Antibodies

Fluorophore conjugated W6/32 and BB7.2 antibodies were purchased from Biolegend. Unconjugated BB7.2 antibodies were purified in at Cell Services core at the University of Pennsylvania.

Lentiviral Production

Lentivirus was produced by co-transfection of Lenti-X 293T cells (Takara Bio) with pSFFV transfer vector containing gene of interest along with psPAX2 packaging vector and pMD2.G envelope vector. Transfections were performed using lipofectamine 3000 according to manufacturer instructions. Virus containing supernatant was then collected each day for up to 3 days and concentrated using Lenti-X concentrator (Takara Bio) according to manufacturer instructions.

Lentiviral Transductions

For lentiviral transductions of tumor cell liens, 100,000 cells were plated per well in 12 well plates in 500µL of advanced RPMI 1640 media supplemented with 10% Premium Plus Heat Inactivated Fetal Bovine Serum, 1% Pen-Strep, 1% GlutaMAX and 1% HEPES (Gibco). Concentrated lentivirus was diluted in 500µL of the same media and added to the cells such that the final volume per well was 1 mL. After 24 hours, transduced cells were supplemented with an additional 1mL of media. Transduction efficiency was determined 48 hours post transduction via intracellular FLAG staining using a PE conjugated anti-DYKDDDDK Tag Antibody (L5, Biolegend). PE MFI was measured via flow cytometry.

CRISPR-Cas9 Editing

721.221 cells expressing a single HLA-I allele were edited by electroporation of CRISPR-Cas9 RNPs. Cas9 was complexed with *TAPBP* specific sgRNA 5'-GAACCAACACUCGAUCACCG-3' (Synthego/Editco) at a 1:3 ratio. Cas9/sgRNA and electroporated using a Lonza 4D-Nucleofector® X Unit program EH-100 and buffers from p3 primary cell 4d-nucleofector® x kit (Lonza). RNPs were prepared by adding in the following order: 5µl P3 Buffer, 1.2 µL sgRNA (100 µM), 2 µL Cas9 (20 µM) per reaction. 8.2 µL of this mixture were added to 200,000 cells resuspended in 15 µL P3 buffer. Reactions were performed in 16 well strips, taking care that liquid fully cover the bottom of wells and that no bubbles were introduced.

Knock out of TAPBP was assessed via Western Blot. 500,000 cells were lysed in 50µL of digitonin solution (1% digitonin in 20mM Tris-HCl 150mM NaCl pH 7.5) containing Halt protease inhibitor cocktail (Thermo Scientific) for 30 minutes at 4C with gentle agitation. Cell solutions were then centrifuged at 12,000g for 30 minutes. Cell lysate was reduced by adding 7µL of LDS sample buffer containing Sample Reducing Agent (Invitrogen) and boiled at 97C for 5 minutes. 20µL of reduced lysate was loaded onto a 4-12% Bis-Tris gel (Invitrogen) and run at 200V for 35 minutes. Gels were transferred to nitrocellulose membranes (Biorad) according to the XCell II Blot Module manual (Invitrogen). Membranes were blocked in TBS-T buffer (25mM Tris, 150mM sodium chloride, 0.05% Tween-20) with 5% milk powder for one hour with gentle agitation. Following blocking, membranes were stained in the same 5% milk TBS-T buffer with 0.5µg/mL of anti-tapasin (7F6, Millipore) or ant-vinculin (W18245A, Biolegend) antibodies overnight at 4C with slight agitation. The following day, membranes were washed in TBS-T buffer 6 times for 5 minutes each with slight agitation. Membranes were then stained with 1:5,000 dilution of HRP-conjugated anti Rat IgG antibody (Thermo Scientific) in 5% milk TBS-T buffer for 1 hour with slight agitation. Membranes were washed again as described above, and visualized using the Pierce ECL Western kit (Thermo Scientific).

Flow Cytometry

For staining, cells were counted and pelleted at 300g 5-10 min at 4C. 100 µL of cell suspension were aliquoted into FACS tubes (BD Biosciences) or 96 well plates then stained with live dead stain such as Live/Dead Violet resuspended in accordance with manufacturer instructions and diluted 1:2000. Cells were stained for at least 10 minutes at room temperature followed by washing. Antibodies were then diluted in FACS buffer (2.5% FBS in 1X PBS with 0.02% sodium azide. 100uL of the antibody mixture were added to each condition and cells resuspended. Cells

were stained for 30 min in the dark at 4C or on ice. Cells were then washed with FACS buffer and then fixed with Fix and Perm Medium A (Thermo Fisher) for 20-30 minutes at room temperature. After 2x washes, cells were resuspended in FACS buffer for subsequent acquisition. For intracellular staining, cells were permeabilized and stained with and antibodies diluted in Fix and Perm Medium B (Thermo Fisher). Cells were then washed twice before acquisition.

HLA-I Trafficking Studies

30,000 Ebc1 cells engineered with or without tapasin constructs were plated in RPMI + 10% FBS and treated with either cycloheximide (20 µg/mL; Millipore Sigma #66-81-9), epoxomicin (100 nM; Millipore Sigma #324801), bafilomicin A1 (100 nM; Millipore Sigma #SML1661), brefeldin A (1x; 5 µg/mL; Biolegend #420601) or vehicle (DMSO) for up to 24 hours. After incubation at 37C, cells were harvested at various time points between 0 and 24 hours. Cells were trypsinized, washed with PBS and stained with LIVE/DEAD Fixable Near-IR stain (Invitrogen #L10119) or LIVE/DEAD Violet Fixable Dead Cell Stain (Invitrogen #L34964) for 10 minutes at 4C followed by a wash with FACS buffer (PBS with 2.5% FBS and 0.02% sodium azide). Cells were subsequently stained with PE anti-HLA-A2 antibody (Biolegend #343306) for 30 minutes at 4C. After staining, cells were washed and fixed in Fixation Medium A (Invitrogen #GAS001S5) before analysis through flow cytometry.

HLA internalization assays

Ebc1-CFP cell lines were harvested and stained with purified HLA-A2 antibody in ice cold RPMI + 10% FBS for 40 mins at 4C. After washing, cells were resuspended in ice cold RPMI + 10% FBS and kept at 4C to suspend HLA internalization. At respective time points, cells were transferred to 37C media to re-initiate HLA internalization process. After all internalization time points were completed, cells were spun down and washed with PBS. Cells were stained with LIVE/DEAD Fixable Near-IR stain (Invitrogen #L10119) for 10 minutes at 4C before being washed with ice cold FACS buffer and fixed (Invitrogen #GAS001S5). A secondary antibody stain (PE goat anti-mouse IgG, Biolegend #405307; APC anti-Flag, Biolegend #637307) was carried out for 20 minutes at RT before cells were washed once more and analyzed through flow cytometry.

Super-Resolution Microscopy and Single Molecule Tracking

The day before imaging,100,000- 150,000 tumor cells were plated on a 35mm glass bottom dish (Cellvis). Cells were stained with a 1:200 dilution of Alexa Fluor 647 conjugated anti-HLA-A2 antibody (BB7.2, Biolegend) in 1 mL of DPBS + calcium chloride + magnesium chloride (Gibco)

and 2% BSA (Miltenyi Biotec) for 25 minutes. Prior to imaging, cells were washed with the PBS + BSA buffer and covered in 1mL of PBS buffer containing 10% w/v Dextrose (Fisher Scientific), 50mM Tris-HCl (Thermo Scientific), 10mM NaCl (Millipore Sigma), 500µg/mL glucose oxidase (Millipore Sigma), 40µg/mL glucose catalase (Millipore Sigma), and 35µL of 1M MEA. Glucose oxidase and catalase enzymes were added to the buffer just prior to imaging.

Single molecule imaging experiments were performed on a custom HILO microscope built on an Olympus TI-83 inverted microscope. A 60X/1.50 NA oil immersion TIRF objective (UplanApo, Oil HR) was used for all experiments, Data were collected using a EMCCD camera (Andor, iXon ultra 897, EM gain 100, exposure time 0.075s). The incubation chamber was humidified and maintained at 37 C with 5% CO2. Four imaging lasers (405 (OBIS), 488 (coherent), 561nm (MPB), 640 nm (MPB) modulated by acousto-optic Tunable Filters (AA Opto-Electronic, France) were combined, aligned, expanded, and focused on the back focal plane of the objective amotorized translation stage was used to adjust the incident angle to achieve highly inclined and laminated optical sheet illumination. A quadband dichroic mirror (zt405/488/561/640) separated the excitation from emission light. The emission from 640 nm excited Alexa647 (21mW at the objective) fluorophores was further filtered with a bandpass filter (705/72, Chroma) in front of the camera. The microscope and camera were controlled using micromanager.

Single molecule localization microscopy (SMLM) analysis (figure 4A) was performed using INSIGHT software (Zhuang lab, Harvard). Single -molecule identification parameter for 2D Gaussian PSFs were set with following parameters (Gaussian height >250 counts, width (250-650) nm, ROI: 7X7 pixels). All single molecule localizations were rendered as 2D Gaussian whose width is weighted by the inverse square root of integrated counts. Single molecule coordinates were generated from photon weighted averaged localization of molecules within 7 pixels in consecutive frames and were used for clustering analysis using DBSCAN with minimum points=50 and distance threshold=0.2 um (Figure 4B). Single molecule tracking (SMT) was performed using trackit (73) with following setting (ROI around cell from max projection, threshold=1, linking distance=7 pixels, minimum track length=3, blinking frames=0). For diffusion mapping (Figure 4C), tracks longer than 10 frames were filtered and mean squared displacement (MSD) of each track for each lag time was computed. First four points of MSD vs lag time was fitted with 2D random diffusion model to extract diffusion coefficients (D). The diffusion distribution was also confirmed and validated using State Array based Single Particle Tracking which considers a range of diffusion coefficients (0.001 um^2/s to 1 um^2/s) and produces an average posterior occupancy for each diffusion coefficient for trajectories (Supplementary figure 4B). For

linearity analysis, immobile jumps (steps <0.2 um) were first filtered and angle between consecutive jumps were computed. Linearity metric was the calculated as the ratio of proportion of angles in range (0°±30°) and (180°±30°) to (90°±30°) and (270°±30°).

DNA-PAINT

Cells were plated on glass bottom dishes and labeled with HLA-A2 antibody (clone BB7.2) and anti-FLAG superclonal antibody mixture (Thermo Fisher # 710662) for 30 minutes on ice in 1x PBS containing calcium and magnesium to prevent cells from lifting off the dish (Thermo Fisher # 14040133) and supplemented with 2% BSA. Cells were then fixed in 4% PFA in 1x PBS for 20 minutes at room temperature. Cells were then washed thoroughly and incubated with single domain secondary antibodies conjugated to donor strands and prepared for imaging according to manufacturer instructions (Massive Photonics).

For analysis of DNA-PAINT data were imported and analyzed using Insight as above. The reconstructed images were first drift corrected using fiducials and rendered as 2-D gaussians with a localization precision of 20 nm. For paired correlation analysis, 20 regions of interest of 5 μm radius were randomly chosen and cross-correlation functions were calculated as described in (32).

T cell Activation, Transduction and Purification

CD8+ T cells were obtained from the Human Immunology Core of the University of Pennsylvania. T cells were then stimulated with anti-CD3/anti-CD28 Dynabeads at a 1:1 ratio in complete RPMI medium supplemented with 10 ng/mL IL-2 for 24 hours prior to transduction. T cells were then replated on tissue culture treated plates coated with 50 μg/mL retronectin (Takara Bio) along with lentivirus. After an additional 48 hours of culture, dynabeads were removed and cells were expanded by adding fresh media supplemented with IL-2. Cells were expanded for several days before purification. 1G4 purification was performed by labeling transduced cells with NYESO with PE-conjugated, HLA-A01*01 tetramer refolded with $NYESO_{157\text{-}165}$ followed by magnetically activated cell sorting (MACS) using anti-PE microbeads.

T cell Killing Assays

T cell killing was assessed via a real time impedance-based assay using the xCELLigence RTCA eSight (Agilent). Target tumor cells (25,000 per well in 100μL media) were plated on E-Plate VIEW 96 well plates (Agilent) and allowed to adhere over 18–24 hours in a 5% CO_2 37°C incubator. T

cells were then added at varying E:T ratios (2:1, 1:1, 1:2, 1:4, 1:8) and T cell killing was monitored via impedance measurements every 15 minutes over a 24-72 hour period. Cell Index (CI) values were automatically calculated by the RTCA eSight software and were normalized to the time point of T cell addition. Percent Killing was calculated using the following equation: % killing =

$$\% Killing = \frac{(CI^{no\ effector} - CI^{effector})}{CI^{no\ effector}} x100$$

For flow cytometry-based T cell killing assays, tumor cells were labeled with CellTrace Violet (Thermo Fisher) and incubated overnight before addition of T cells. After overnight co-culture, cells were trypsinized, harvested and stained with LIVE/DEAD reagents at a 1:2000 dilution for 10 minutes on ice. Cells were then fixed and washed twice with FACS buffer. CountBright beads were then added to determine the relative number of surviving tumors cells.

Isolation of HLA ligands by immunoaffinity purification

HLA class I molecules were isolated using standard immunoaffinity purification methods as previously described (*8*) with minor mortification. In brief, cell pellets were lysed in 10 mM CHAPS/PBS (Millipore Sigma) containing 1× protease inhibitor (Complete; Roche) and Phosphatase Inhibitor cocktail (Roche). HLA molecules were purified overnight using the HLA-A02-specific monoclonal antibody (BB7.2) covalently linked to CNBr-activated sepharose (GE Healthcare). pMHC complexes were eluted by the repeated addition of 0.2% trifluoroacetic acid (Merck). Elution fractions E1–E4 were pooled, and free MHC ligands were isolated by ultrafiltration using 5kDa centrifugal filter units (Human Metabolome Technologies America, Inc). Extracted peptides were lyophilized to approximately 30-50 µl using a lyophilizer (Labconco) then stored at –80 °C. Samples were desalted by C18 stage tip. Eluted peptides were dried by vacuum centrifugation and reconstituted in 0.1% triflouroacetic acid/0.015% n-Dodecyl-beta-D-maltoside ((DDM)-Sigma) containing iRT peptides (Biognosys Schlieren, Switzerland) and stored at –80 °C until analysis by LC–MS/MS.

For Figure 8, cells were lysed on ice at a density of $5x10^7$ cells per ml with 0.5% IGEPAL CA-630, 0.25% sodium deoxycholate, 1 mM EDTA, 0.2 mM iodoacetamide, 1 mM PMSF, Roche Complete Protease Inhibitor Cocktail in PBS. Lysates were cleared by centrifugation at 21,000g for 30 min, 4°C. HLA class I complexes were purified with monoclonal anti-HLA class I (clone W6/32) antibody-coupled resin incubating cell lysate overnight. The resin was washed once with lysis buffer, followed by buffer A (150 mM NaCl, 20 mM Tris, pH 7.4), buffer B (400 mM NaCl, 20 mM Tris, pH 7.4), a second buffer A wash and finally with buffer C (20 mM Tris, pH 8.0). Peptides were eluted from HLA complexes in 0.1 M acetic acid. Peptides were further purified with C18 resin and eluted in 30% acetonitrile, 0.1% trifluoroacetic acid.

Analysis of HLA ligands by LC–MS/MS

LC-MS data were acquired on a nanoElute 2 system connected to a timsTOF Ultra 2 using data-dependent Parallel Accumulation and Serial Fragmentation (DDA-PASEF) mode. Peptides were separated on an IonOpticks 25 cm × 75 µm Aurora Elite C18 column, maintained at 50°C.
For Figure 8, LC-MS data were acquired on a timsTOF Ultra using similar DDA-PASEF methods.

LC–MS/MS

LC-MS/MS data were acquired using a nanoElute 2 system coupled to a timsTOF Ultra 2. Peptides were separated on a 25 cm × 75 µm Ionopicks Aurora Elite C18 column, maintained at 50°C. The flow rate was set to 350 nL/min, with mobile phase B increasing from 5% to 23% over 40 minutes, then from 23% to 35% over 20 minutes, followed by a ramp to 90% B over 1 minute, and held at 90% B for 9 minutes.
Data were acquired in DDA-PASEF mode on the timsTOF Ultra 2. MS1 scans covered a range of 100–1700 m/z with an ion mobility window of 1/K0 = 1.65 Vs/cm^2 to 0.64 Vs/cm^2. Five PASEF ramps were collected with accumulation and ramp times of 100 ms. Precursors exceeding an intensity threshold of 500 were isolated with a 2 Th window for m/z <700 and a 3 Th window for m/z >800, and re-sequenced until a target intensity of 20,000 was reached, with a dynamic exclusion of 40 seconds. The precursor polygon was adjusted to include singly charged precursors from 700–1375 m/z and multiply charged precursors from 272–800 m/z.

System Suitability and Quality Control

The suitability of the instruments was monitored using QuiC software (Biognosys, Schlieren, Switzerland) for the analysis of the spiked-in iRT peptides. Meanwhile, as a measure for quality control, we injected standard K562 (Promega) protein digest before in the middle of, and after sample set using DIA mode. The collected data were analyzed in DIA-NN (78) and the output was subsequently used to track the quality of the instrumentation.

Mass spectrometry raw data processing

The MS/MS raw files underwent processing using MsFragger v.22.0 with MSBooster (35). For this analysis, we employed a reference of the human proteome from UniProt, consisting of Swiss-Prot and TrEMBL entries, supplemented with a list of 245 common protein contaminants. The default parameters in the Nonspecific-HLA workflow were utilized to search the raw data. In summary, protein digestion was set as nonspecific, and both precursor and fragment mass

tolerances were set to 20 ppm. Protein N-terminal acetylation, oxidation of Met, Cysteinylation of Cys, and pyroglutamate of N-terminal Gln/Glu were considered as variable modifications. Percolator with a minimum probability of 0.5 was used for PSM validation. MS1 quantification was performed in IonQuant (75) without conducting a match between runs.

For Figure 8, data was processed with proteoscape (Bruker) software following manufacturer recommended parameters into account with the exception of amino acid modifications, where only Methionine oxidation was considered, finally filtering identifications for 1% FDR.

Multiomics analysis of identified peptides

Transcriptome data (RNASeq) from 224 patient derived neuroblastoma tumors and 39 human neuroblastoma cell lines were used along with immunopeptidomic data derived from the 844 peptides found in tapasin-TM cells but not parental cells. 8-10-mer peptides were pre-filtered based on predicted binding to HLA-A2 using NetMHCpan 4.1. Peptides were mapped to the human genome using MSFragger software. Genes associated with peptides were then used to rank data based on normal tissues expression using the GTEx version 10 database. Ranking was performed using rank() in base R programming language (similar method to the Wilcoxen rank-sum test). The top 100 genes with lowest normal expression were then further filtered to remove genes with low median transcript expression in neuroblastoma (average TPM<5). Data were further refined by removing genes with TPM>20 in any single tissue excluding testis and adrenal gland. Testis was excluded as it is a source of germ line or developmentally related antigens, and adrenal gland was excluded as adrenalectomy is a common surgical procedure to treat neuroblastoma. Remaining genes were then filtered at the peptide level by removal of peptides known to be presented in normal tissue using the HLA-Atlas database. Lastly, peptides mapping to multiple genes were removed and remaining peptides further analyzed with NetMHCpan 4.1 for binding to top 20 common HLA-I alleles by population frequency and presentation in normal and cancerous tissues using HLA-Compass (Alithea Bio).

A similar bioinformatics pipeline was implemented for the immunopeptidomics campaign using W6/32 antibody for HLA-I complex immunoprecipitation. Peptide data was first filtered to only include 8-11 mer peptides that were predicted to bind a HLA-I allele present in the deriving cell line. Following filtering on low normal expression (as above but with TPM<10 thresholds) and high neuroblastoma tumor expression, we again used HLA-Ligand Atlas to remove genes associated with peptides presented in normal tissue excluding testis, thymus, ovary and adrenal gland. We then performed an additional filtering step using the Evo-devo mammalian organ expression

database (50), which consists of transcriptome level expression data for key organs during fetal development and across lifespan. This additional filtering step allowed for identification of putative oncofetal antigens and cancer testis or ovary antigens. Lastly, peptides mapping to multiple genes were removed.

Human DCs generation

DCs were generated by the “adherence method” (79). Briefly, peripheral blood mononuclear cells (PBMCs) were purchased from Human Immunology Core from University of Pennsylvania. A total of 1×10^8 mononuclear cells were plated in RPMI containing 2 mM GlutaMAX-I (gibco, #35050-061)+ 10 mM HEPES (gibco, #15630-080) + non-essential amino-acids (gibco, #1140-050) + Penicillin Streptomycin Solution (corning, #30-002-CI) + 1% AB Human sera not inactivated (Sigma-Aldrich, #H3667) in T-75 flasks for 2 hours at 37 ℃. The nonadherent cells were removed by washing with phosphate-buffered saline (PBS), and the adherent cells were cultured with 100 ng/mL granulocyte-macrophage colony-stimulating factor (GM-CSF) (Peptrotech, #300-03) and 20 ng/mL interleukin-4 (IL-4) (GensScript, #Z02925-10) for 5 days. IL-4 and GM-CSF were replenished on day 2 and 4. On day 5, cells were harvested and concentrated at 1 $x10^6$/mL with 200 ng/mL GM-CSF, 40 ng/mL IL-4 and, for maturation, 100 U/mL IFN-γ (Preprotech, # 300-02), 5µg/mL poly I:C (InvivoGen, #tlrl-pic) and R848 (InvivoGen, #tlrl-r848) with irradiated (10,000 RADS) CD40L-expressing K562 cells.

Human CD8+ T cells priming

Autologous CD8+ T cells were purchased from Human Immunology Core from University of Pennsylvania. Purified CD8+ T cells (5×10^6 cells/ml) were cultured at a 20:1 ratio with irradiated (2500 Rads) autologous mDC pulsed with peptide (40 µg per 1×10^6 DC/ml) in 24-well trays in Optimizer CST media (Gibco) supplemented with 5% pooled human sera. Human IL-7 (10 ng/ml, Preprotech, #200-07)), IL-15 (5 ng/ml, Preprotech, #200-15), and IL-12 (10 ng/ml, Preprotech, #200-12) were added on day 0. Fresh media supplemented with IL-7 (10 ng/ml) and IL-15 (5 ng/ml) was added on day 7. Fourteen days after primary mDC stimulation, T cell cultures were harvested and re-stimulated with irradiated (2500 Rads) peptide-pulsed mDC. Cell culture media was supplemented with 50 U/ml IL-2 (Peprotech, # 200-02) starting day 2 then every 48 h following secondary stimulation. On days 10–14 of secondary mDC stimulation, antigen-specific T cell responses were identified by IFN-γ ELISPOT assay.

Human IFN-γ ELISPOT assay

CD8+ T cell reactivity to peptide antigen was assessed by interferon-γ (IFN-γ) ELISPOT assay. The spot number was determined using the AID ELISPOT reader (AID Autoimmun Diagnostika GmbH, Strassberg, Germany) and AID ELISPOT 7.0 software. A positive response was recorded if the number of spots in the peptide-exposed wells was two times or more higher than the number of spots in the unstimulated wells and if there was a minimum of 20 (after subtraction of background spots) peptide-specific spots per 5×10^5 CD8+ cells.

Recombinant MHC protein expression, refolding and purification

Plasmid DNA encoding the BirA substrate peptide (BSP, LHHILDAQKMVWNHR)-tagged luminal domain of the MHC I heavy chains and human β2-microglobulin (β2m) were provided by the National Institutes of Health (NIH) tetramer facility (Emory University) and transformed into E. coli BL21(DE3) cells (Novagen). BSP-tagged MHC I proteins were expressed by autoinduction in Luria–Bertani medium and inclusion bodies were collected and purified using a standard protocol (9, 23). In vitro refolding of BSP-tagged pMHC I molecules was performed by slowly diluting a 100-mg mixture of BSP-tagged MHC I and β2m at a 1:3 molar ratio in refolding buffer (0.4 M l-arginine, 100 mM Tris pH 8, 2 mM EDTA, 4.9 mM reduced glutathione and 0.57 mM oxidized glutathione) containing 10 mg of the placeholder peptide. BSP-tagged pMHC I refolding proceeded for 96 h and was followed by overnight dialysis against ten liters of SEC buffer (25 mM Tris pH 8.0, 100 mM NaCl). Refolded MHC I complexes were concentrated to 5 mL by tangential flow filtration and injected onto a HiLoad 16/600 Superdex 200 pg column. Fractions containing pMHC I were analyzed by SDS-PAGE and final protein concentrations were determined using a Nanodrop spectrophotometer.

The BSP-tagged pMHC I proteins were biotinylated using the BirA biotin–protein ligase bulk reaction kit (Avidity) according to the manufacturer's instructions and prepared at a final concentration of 2 mg/mL monomer. The level of biotinylation was evaluated by SDS–PAGE gel-shift assay in the presence of excess streptavidin.

Differential scanning fluorimetry was used to assess the thermal stabilities of the refolded pMHC I molecules. 10 μM pMHC I was mixed with 10× SYPRO Orange dye (Invitrogen) in a buffer of 150 mM NaCl and 20 mM sodium phosphate pH 7.4. 20 μL samples were loaded into MicroAmp Optical 384-well plate and the experiment was performed on a QuantStudio 5 real-time PCR machine with excitation and emission wavelengths set to 470 nm and 569 nm. The temperature

was incrementally increased at a rate of 1 °C per minute between 25 and 95 °C. Data analysis and fitting were performed in GraphPad Prism 9.

Supplemental Figures

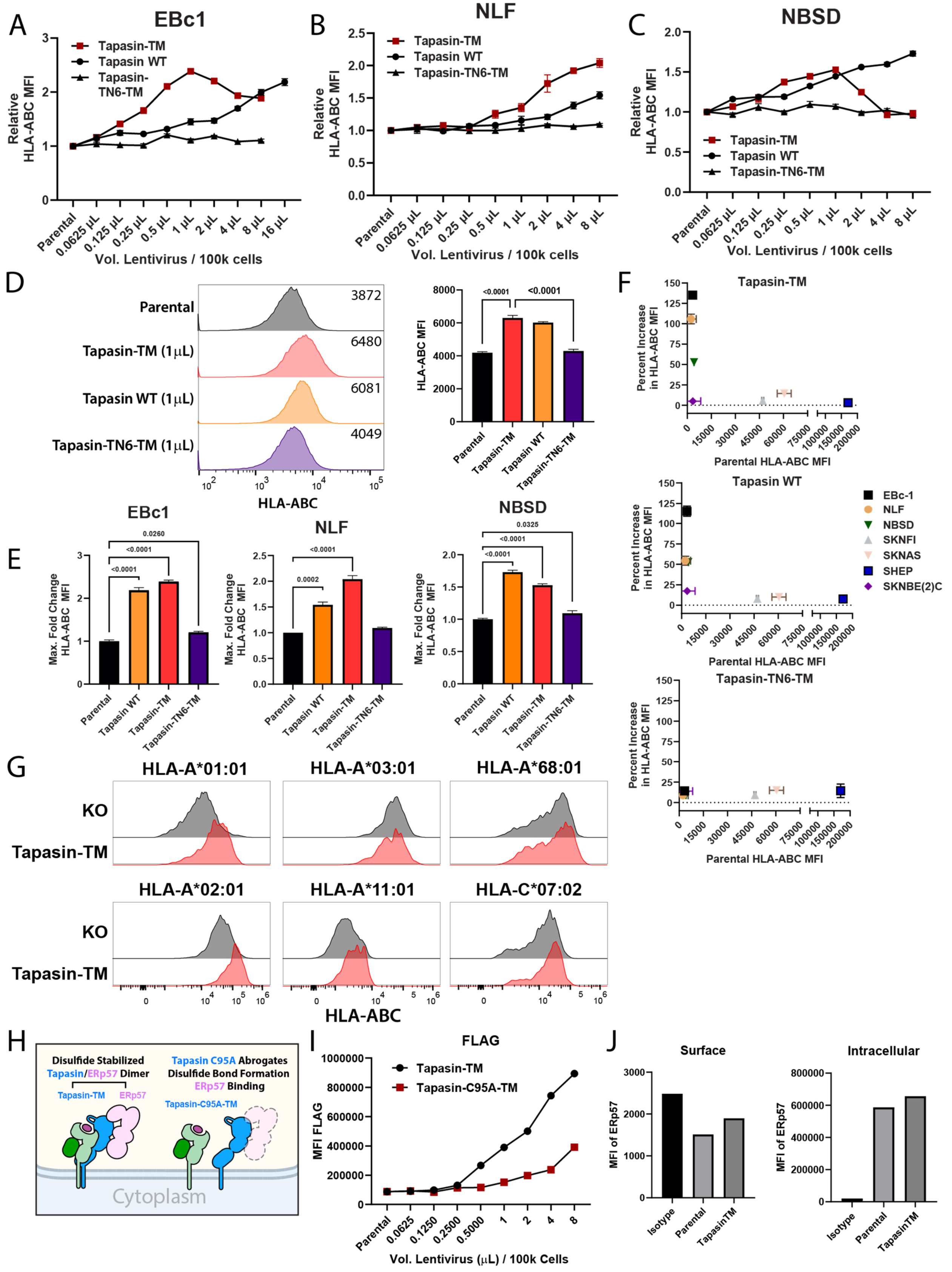

Supplemental Figure 1. Tapasin-TM causes HLA-I upregulation in EBc1, NLF, and NBSD cells in a ERp57 dependent manner. A) EBc1, **B)** NLF and **C)** NBSD cell line was transduced with a titration of concentrated lentivirus and evaluated for HLA-I complex expression by staining with W6/32 antibody. Relative MFI of HLA-I in NBSD cells transduced with tapasin constructs is shown. Data represent 4-8 technical replicates compiled from 2 independent experiments. **D)** Histograms depicting HLA-I expression and quantification of MFI for NBSD cells transduced with 1 µL of different tapasin lentiviruses. **E)** Quantification of max fold change of HLA-I MFI in EBc1, NLF, and NBSD cells. **F)** Maximum percentage increase in HLA-I by W6/32 staining for tested neuroblastoma lines. Negative values were reported as zero. 2-4 technical replicates pooled from 2 independent experiments. **G)** Histograms of HLA-ABC expression in tapasin KO 721.221 monoallelic HLA-I cell lines expressing tapasin-TM. **H)** Schematic depicting the consequence of the C95A mutation. C95 (of the luminal domain of tapasin) forms disulfide bond with ERp57, which stabilizes tapasin. **I)** EBc1 cells were transduced with either tapasin-TM or tapasin-C95A-TM and then evaluated for total FLAG expression by intracellular staining. N=2 technical replicates; data is representative of two independent experiments. **J)** Surface and intracellular staining for ERp57 in parental EBc1 cells and those transduced with tapasin-TM. MFI of ERp57 is shown.

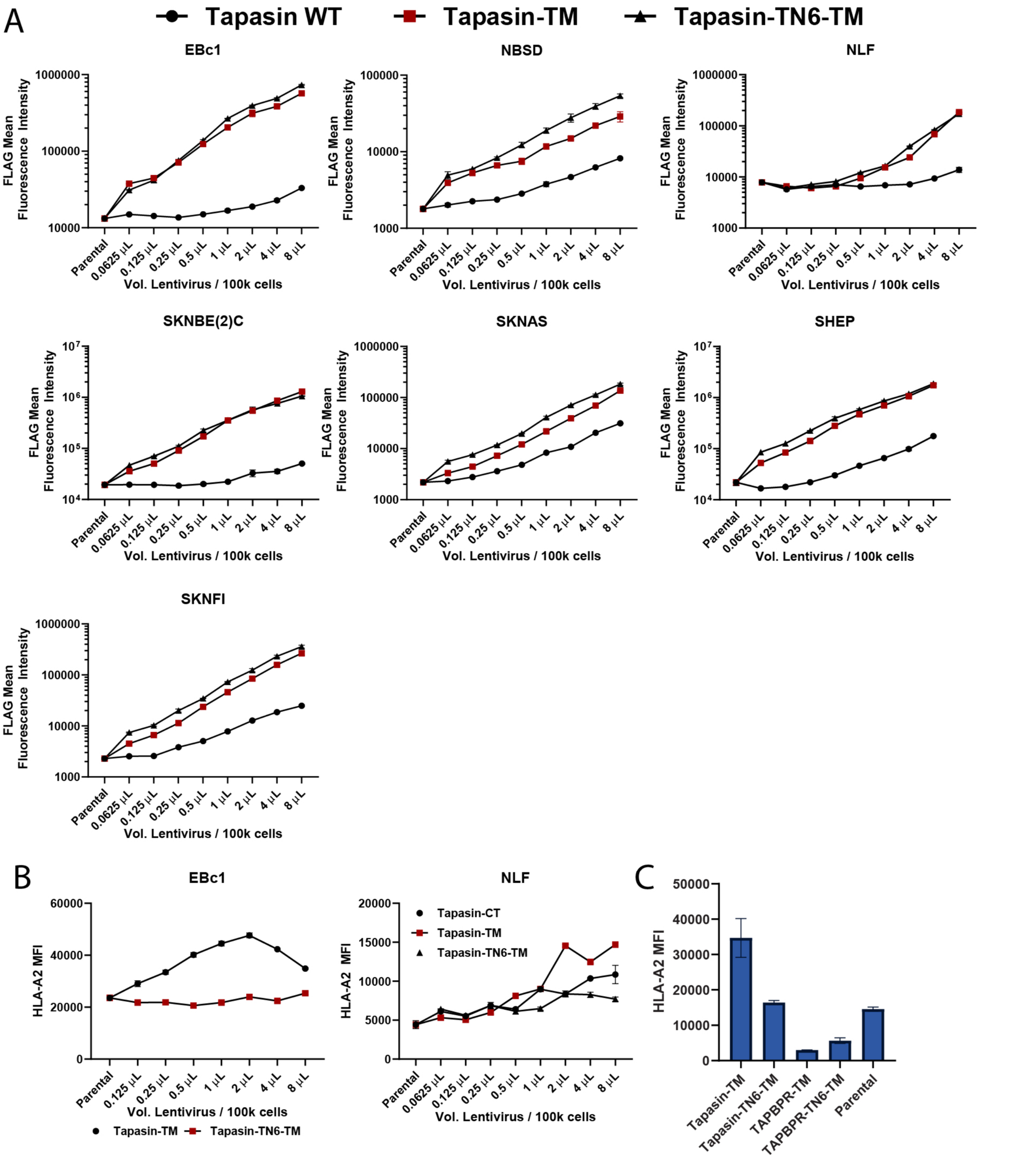

Supplemental Figure 2. Tapasin-TM has higher protein level expression than WT tapasin. **A)** Expression of tapasin constructs as determined by intracellular staining for FLAG followed by flow cytometry analysis. Mean fluorescence intensity is shown. **B)** MFI of surface HLA-A2 (as determined by BB7.2 mAb staining in EBc1 and NLF cells transduced with tapasin variants. **C)** HLA-A2 MFI in EBc1 cells transduced with tapasin-TM and TAPBPR-TM variants. TAPBPR variants have identical design in that their C-terminus is replaced with that of HLA-G.

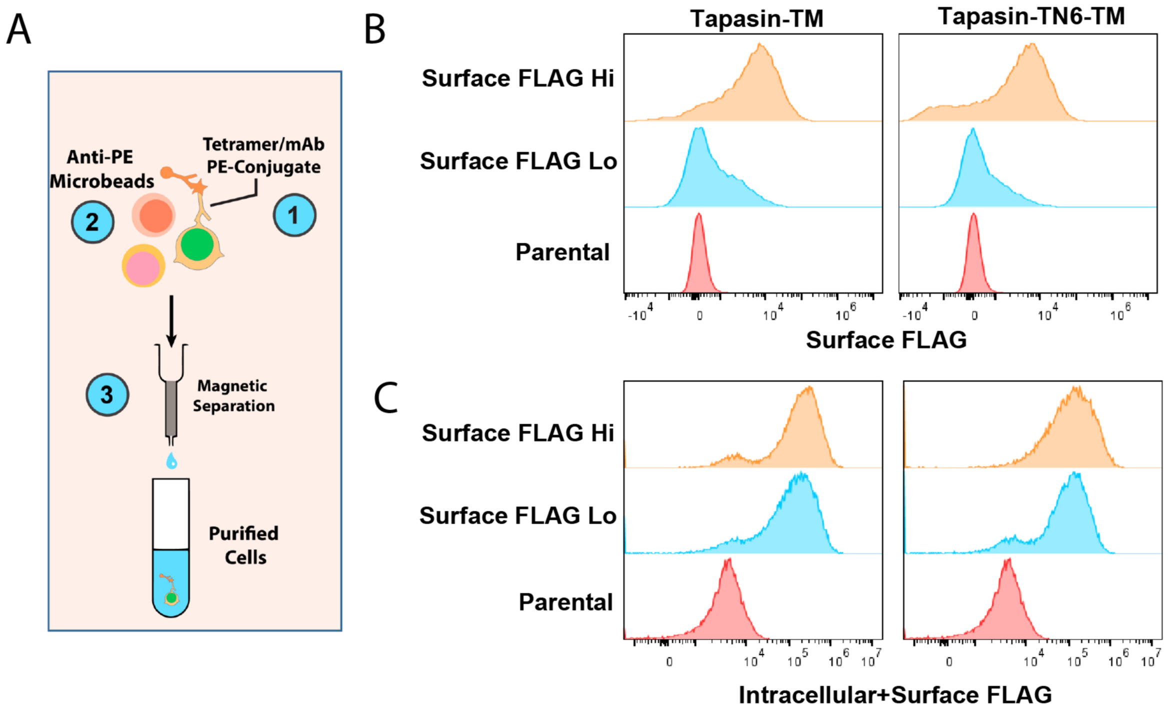

Supplemental Figure 3. Tapasin-TM has varying levels of surface expression in EBc1 cells. A) Schematic depicting method for separating surface tapasin-TM^{Hi} and surface tapasin-TM^{Lo} cells. 1) Tapasin-TM transduced EBc1 cells were labeled with anti-FLAG-tag PE conjugate followed by 2) labeling with anti-PE magnetic beads and 3) magnetic isolation with LS column (Miltenyi Biotec). **B)** Flow cytometry plots depicting surface expression of tapasin-TM and tapasin-TN6-TM as detected by the FLAG-tag following magnetic bead separation. **C)** Intracellular staining for FLAG-tag in surface tapasin-TM^{Hi} and surface tapasin-TM^{Lo} Ebc1 cells showing similar levels of intracellular tapasin-TM and tapasin-TN6-TM despite differences in surface expression.

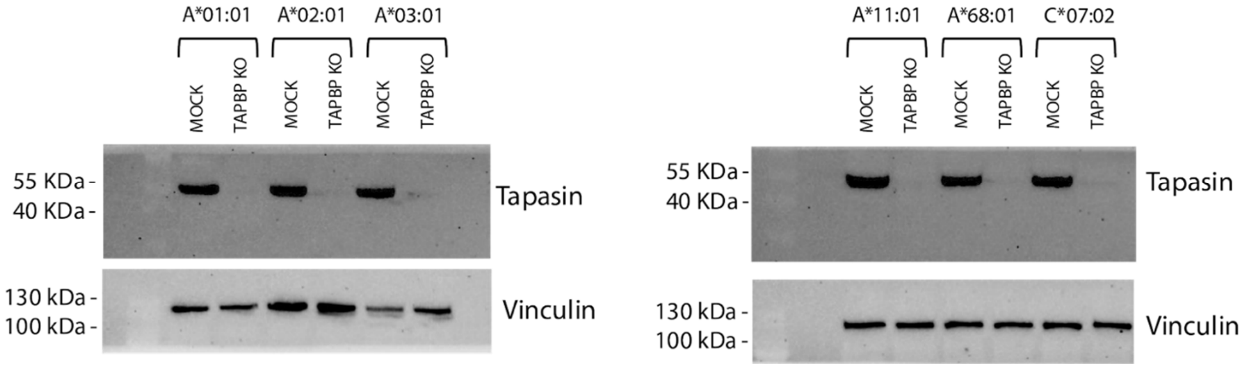

Supplemental Figure 4. CRISPR mediated knock out of *TAPBP* in 721.221 HLA-I monoallelic cell lines. 721.221 HLA-I monoallelic cell lines (HLA-A*01:01, HLA-A*02:01, HLA-A*03:01, HLA-A*11:01, HLA-A*68:01, HLA-C*07:02) were electroporated with Cas9/ *TAPBP* sgRNA (tapasin) or PBS. Knock out of *TAPBP* was assessed via western blot. Presence of tapasin and loading control vinculin was determined by staining with anti-tapasin or anti-vinculin antibodies and an HRP-conjugated secondary antibody. Membranes were visualized using the Pierce ECL Western kit.

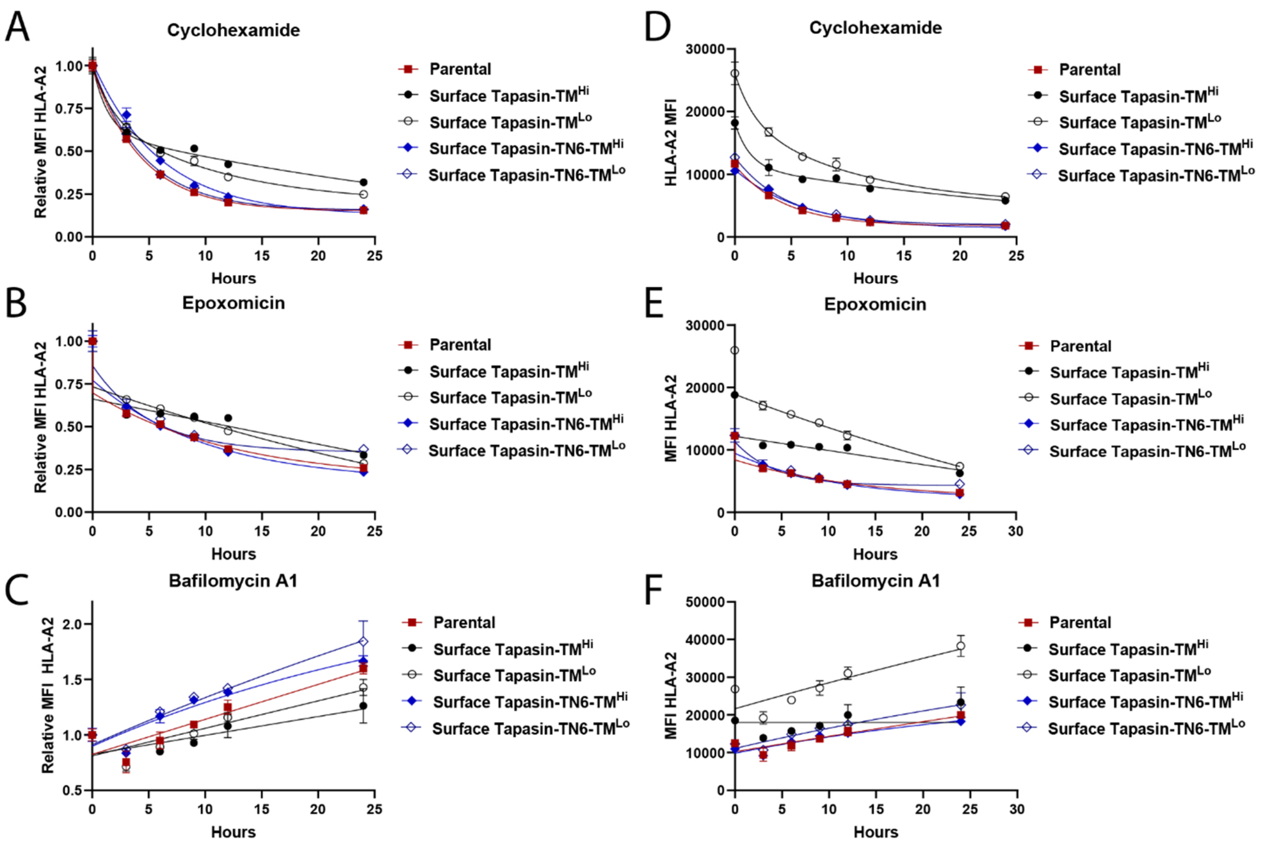

Supplemental Figure 5. Surface Tapasin-TM positively regulates HLA-I stability. A-F) Ebc1 parental or tapasin-TM and tapasin-TN6-TM transduced cells sorted into surface tapasin high and low expressors were treated with cycloheximide (20 µg/mL), brefeldin A (1x; 5 µg/mL), bafilomycin A1 (100 nM) and epoxomicin (100 nM) or vehicle (DMSO) for up to 24 hours followed by flow cytometry analysis for surface folded-HLA-A2 expression (BB7.2 antibody staining). For cycloheximide, brefeldin A and epoxomicin, curves represent two-phase exponential decay regression performed in Prism. For bafilomycin A1, two-phase association regression is shown. **A)** Relative MFI HLA-A2 for cells treated with cycloheximide, **B)** epoxomicin and **C)** bafilomycin A1. **D-F)** Raw HLA-A2 MFI for corresponding to data shown in A-C.

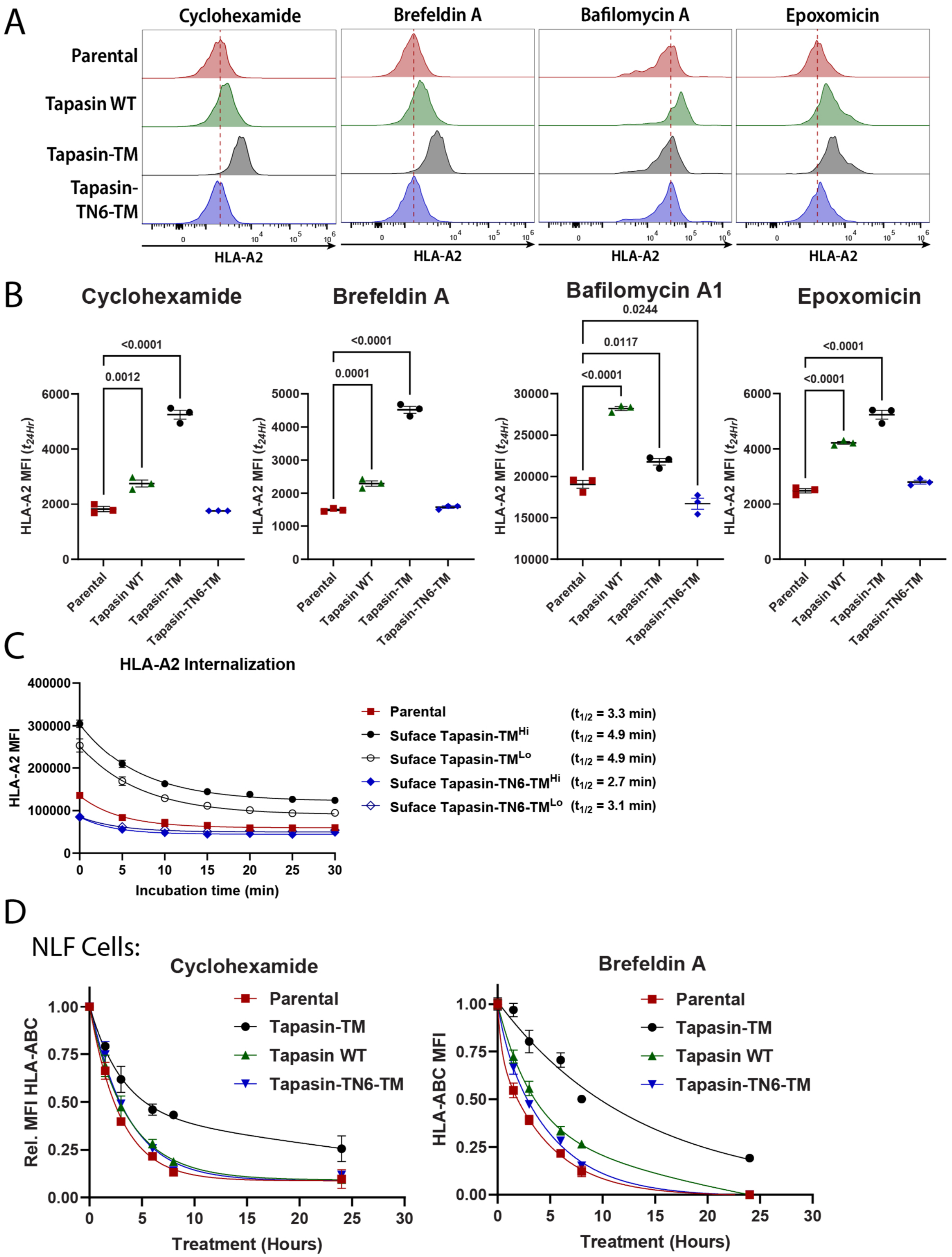

A
Cyclohexamide
Brefeldin A
Bafilomycin A
Epoxomicin
Parental
Tapasin WT
Tapasin-TM
Tapasin-TN6-TM
HLA-A2
B
Cyclohexamide
Brefeldin A
Bafilomycin A1
Epoxomicin
HLA-A2 MFI (t_{24Hr})
<0.0001
0.0012
0.0001
0.0244
0.0117
Parental
Tapasin WT
Tapasin-TM
Tapasin-TN6-TM
C
HLA-A2 Internalization
HLA-A2 MFI
Incubation time (min)
Parental ($t_{1/2}$ = 3.3 min)
Suface Tapasin-TM^{Hi} ($t_{1/2}$ = 4.9 min)
Suface Tapasin-TM^{Lo} ($t_{1/2}$ = 4.9 min)
Suface Tapasin-TN6-TM^{Hi} ($t_{1/2}$ = 2.7 min)
Suface Tapasin-TN6-TM^{Lo} ($t_{1/2}$ = 3.1 min)
D
NLF Cells:
Cyclohexamide
Brefeldin A
Rel. MFI HLA-ABC
HLA-ABC MFI
Treatment (Hours)
Parental
Tapasin-TM
Tapasin WT
Tapasin-TN6-TM

Supplemental Figure 6. Tapasin-TM enhances HLA-I stability. A) Histograms depicting HLA-A2 surface expression in EBc1 cells treated with cycloheximide, brefeldin A, bafilomycin A1 and epoxomicin for 24 hours. **B)** Quantification of MFI HLA-A2 for A. **C)** Antibody-based HLA-A2 internalization assay. Parental Ebc1 cells and those expressing high and low surface tapasin-TM and tapasin-TN6-TM were labeled with anti-HLA-A2 antibody (BB7.2) on ice prior to incubation for different periods at 37C to allow for antibody internalization. Remaining surface antibody was then probed using a fluorophore conjugated secondary antibody. MFI of HLA-A2 over 30 minutes is shown. Half-life data are derived from one-phase decay regressions. **D)** Decay of HLA-I in NLF cells treated with cycloheximide and brefeldin A for the indicated time period. Cells were stained with W6/32 antibody to stain for HLA-ABC and evaluated by flow (n=3-12 technical replicates are shown). MFI values for each cell line were normalized to the parental condition.

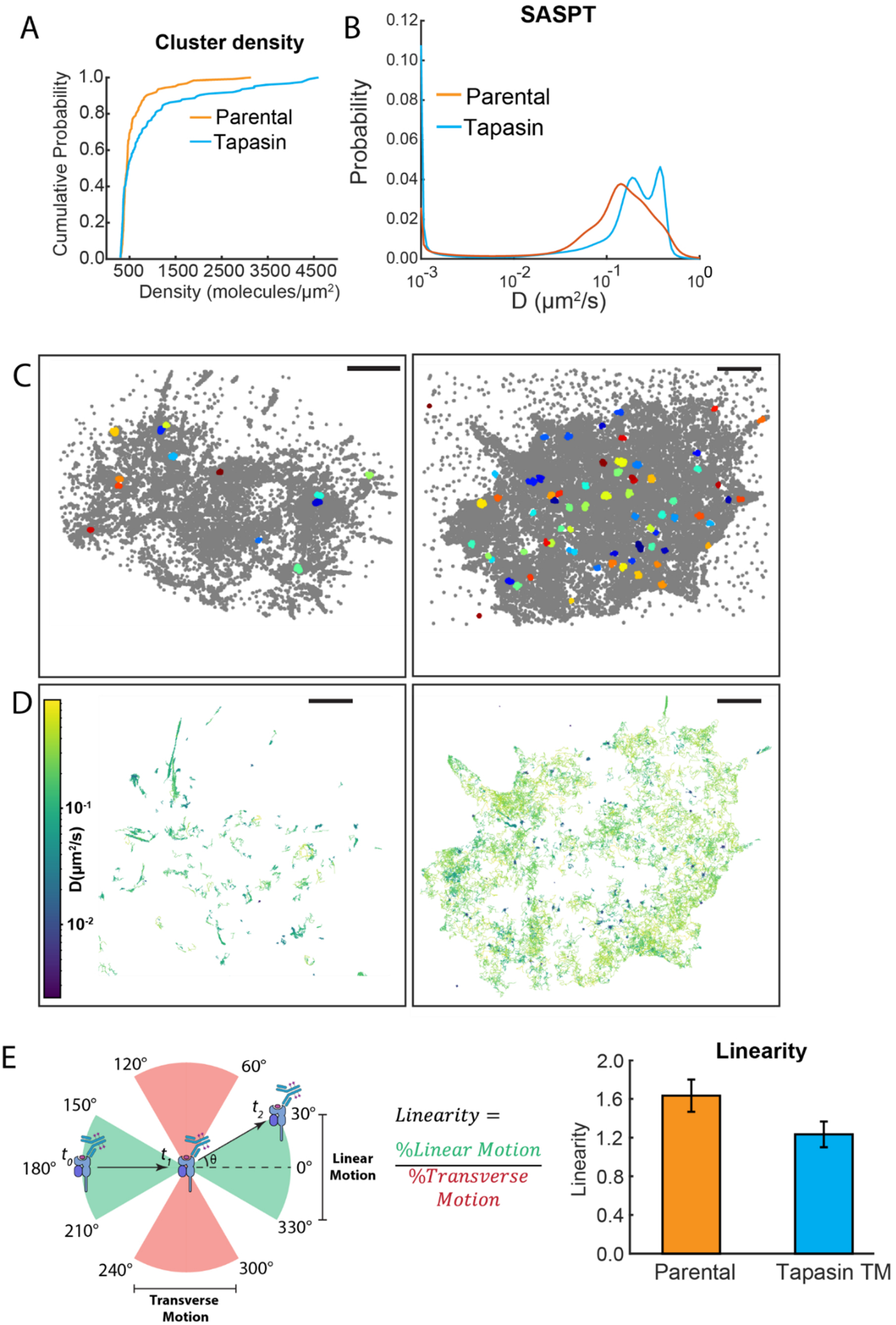

A
Cluster density
Cumulative Probability
Parental
Tapasin
Density (molecules/μm²)
B
SASPT
Probability
D (μm²/s)
C
D
D(μm²/s)
E
Linear Motion
Transverse Motion
Linearity =
%Linear Motion
%Transverse Motion
Linearity
Parental
Tapasin TM

Supplemental Figure 7. Cumulative distribution function (CDF) of cluster density from identified clusters and state array for single particle tracking (SASPT) analysis. Representative images for clustering and diffusion analyses. A) Cumulative distribution function (CDF) of cluster density from identified clusters. **B)** State array for single particle tracking (SASPT) analysis of single molecule tracks revealing occupancy probability of diffusion coefficients of Parental and Tapasin-TM. **C)** Clusters of local high density regions detected using DBSCAN in tapasin-TM (left) and parental (right). **D)** Diffusion co-efficient mapping of tracks in tapasin-TM (left)and parental (right). **E)** Schematic and quantification of linearity between parental and tapasin-TM. Scale bar: 5 um, zooms: 100 nm. Linearity is calculated as ratio of angles Ө exhibiting linear vs. transversion motion.

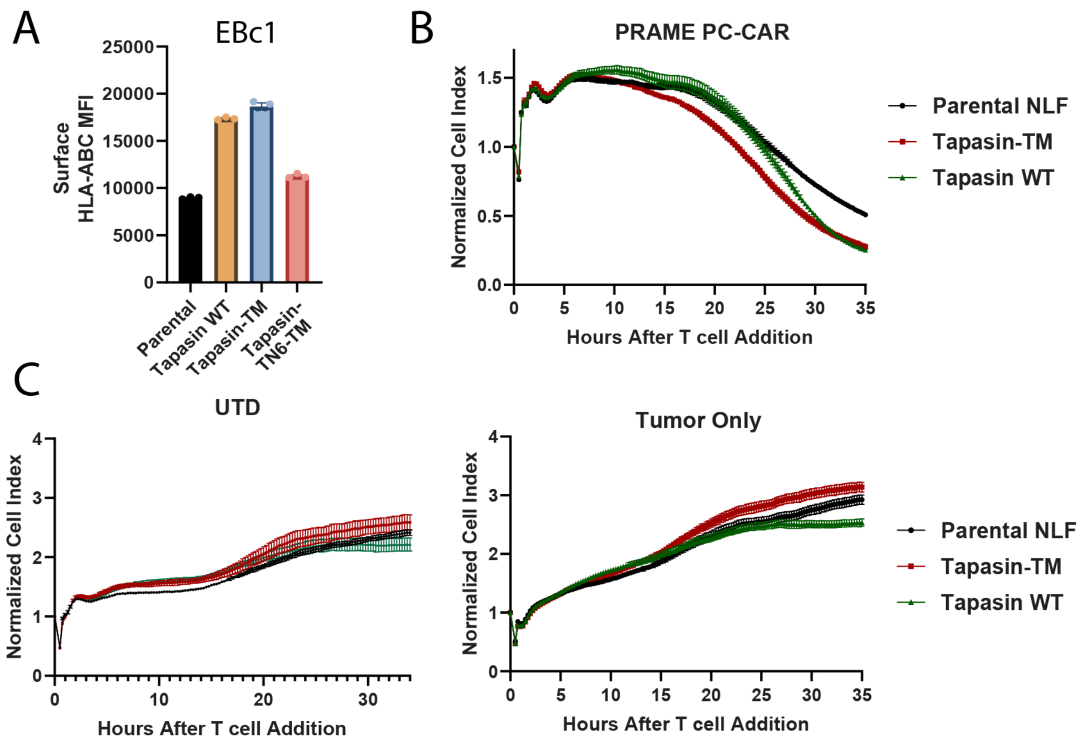

Supplemental Figure 8. Tapasin-TM enhances killing of NLF cells by HLA-A2/PRAME PC-CAR T cells. A) MFI of surface HLA-ABC as measured by flow cytometry in cells transduced with tapasin variants. NLF cells were plated on gold electrode coated tissue culture plates and allowed to adhere for ~2 days. HLA-A2/PRAME specific PC-CAR T cells were then added at a 2:1 E:T ratio and normalized cell index monitored. **B)** Normalized Cell Index for NLF cells co-cultured with PRAME-PC-CAR T cells **C)** or alone or with "Untransduced" T cells which were activated and processed in parallel but not transduced.

Samples:
1-3: Parental **4-6:** Tapasin WT **7-9:** Tapasin-TM **9-12:** Tapasin-TN6-TM

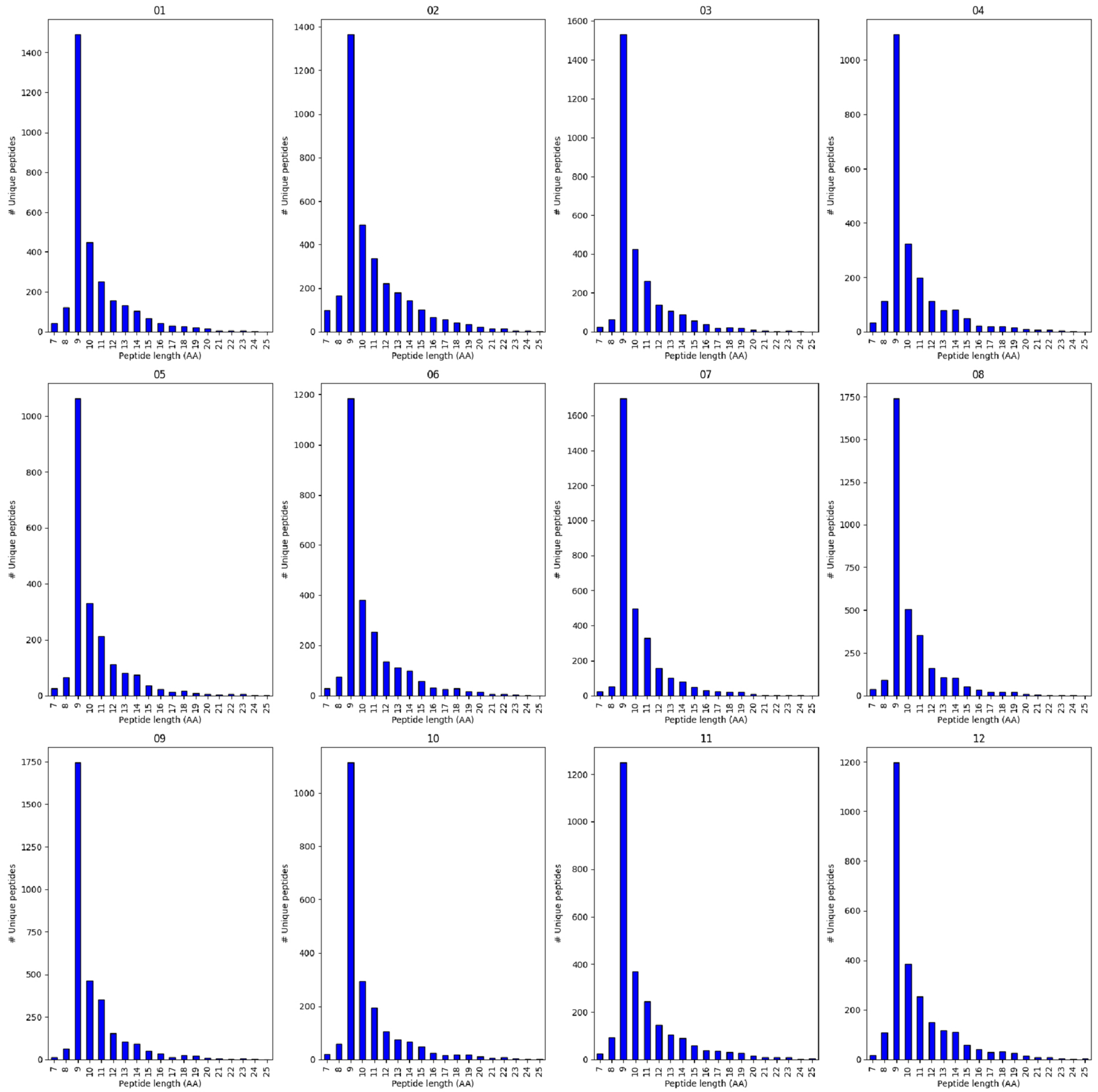

Supplemental Figure 9. Peptide length distribution of peptides derived from HLA-A2 molecules from EBc1 cells expressing tapasin variants.

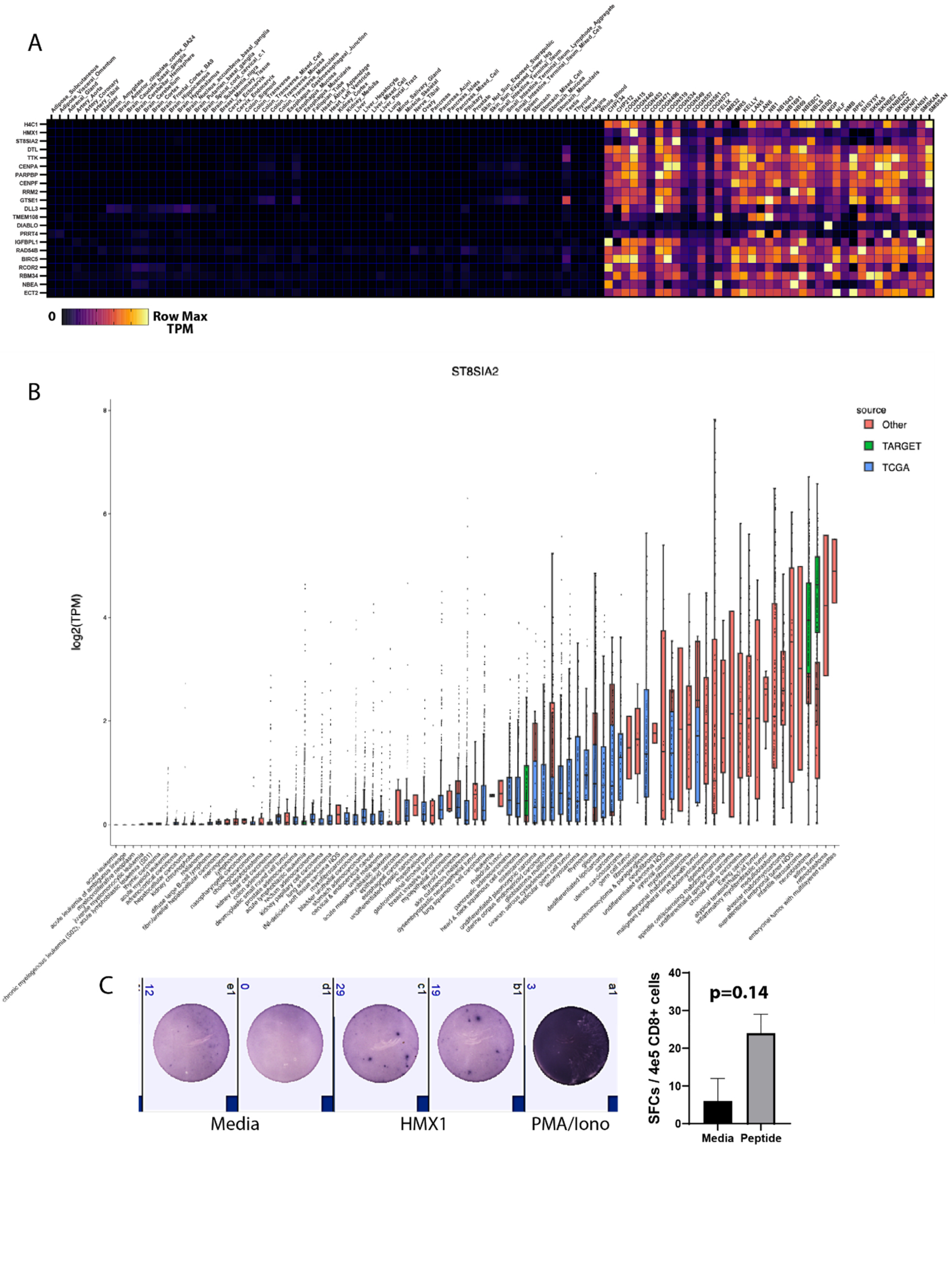
A
H4C1
HMX1
ST8SIA2
DTL
TTK
CENPA
PARPBP
CENPF
RRM2
GTSE1
DLL3
TMEM108
DIABLO
PRRT4
IGFBPL1
RAD54B
BIRC5
RCOR2
RBM34
NBEA
ECT2
0
Row Max
TPM
ST8SIA2
B
log2(TPM)
source
Other
TARGET
TCGA
C
Media
HMX1
PMA/Iono
p=0.14
SFCs / 4e5 CD8+ cells
Media
Peptide

Supplemental Figure 10. Identification of bona fide tumor-associated antigens in neuroblastoma cells using HLA-shuttle. Peptides identified in tapasin-TM expressing EBc1 cells but not in the parental cell line were further analyzed for the presence of clinically relevant targets. **A)** Transcriptome data (RNASeq) from 39 human neuroblastoma cell lines were used along with immunopeptidomic data derived from the 844 peptides found in tapasin-TM cells but not parental cells. Data were analyzed for presence of TAAs with suitable therapeutic profile. All peptides were pre-filtered based on predicted binding to HLA-A2 using NetMHCpan 4.1. Peptides were mapped to the human genome using MSFragger software. Genes associated with peptides were then used to rank data based on normal tissues expression using the GTEx version 10 database. Ranking was performed using a similar method to the Wilcoxen rank-sum test. The top 100 genes with lowest normal expression were then further filtered to remove genes with low median transcript expression in neuroblastoma (expressed in <50% of cell lines and TPM<5 were removed). Data were further refined by removing genes with TPM>20 in any single tissue excluding testis and adrenal gland. Testis was excluded as it is a source of germ line or developmentally related antigens, and adrenal gland was excluded as adrenalectomy is a common surgical procedure to treat neuroblastoma. TPM for neuroblastoma lines was collapsed to a single Remaining genes were then filtered at the peptide level by removal of peptides known to be presented in normal tissue using the HLA-Atlas database. **B)** Expression of ST8SIA2 across tumors. Data is derived from TCGA, TARGET and in-house databases. **C)** IFN-γ ELISPOT for human CD8 T cells co-cultured with autologous PBMC-derived DCs pulsed with the HMX1 peptide, FLIENLLAA. Cells stimulated with 5 μg/mL PMA/Ionomycin mixture was used as a positive control and cells treated with media served as negative control. Number of spot forming cells (SPCs) per 400,000 cells is shown. Statistics were calculated by unpaired t test.

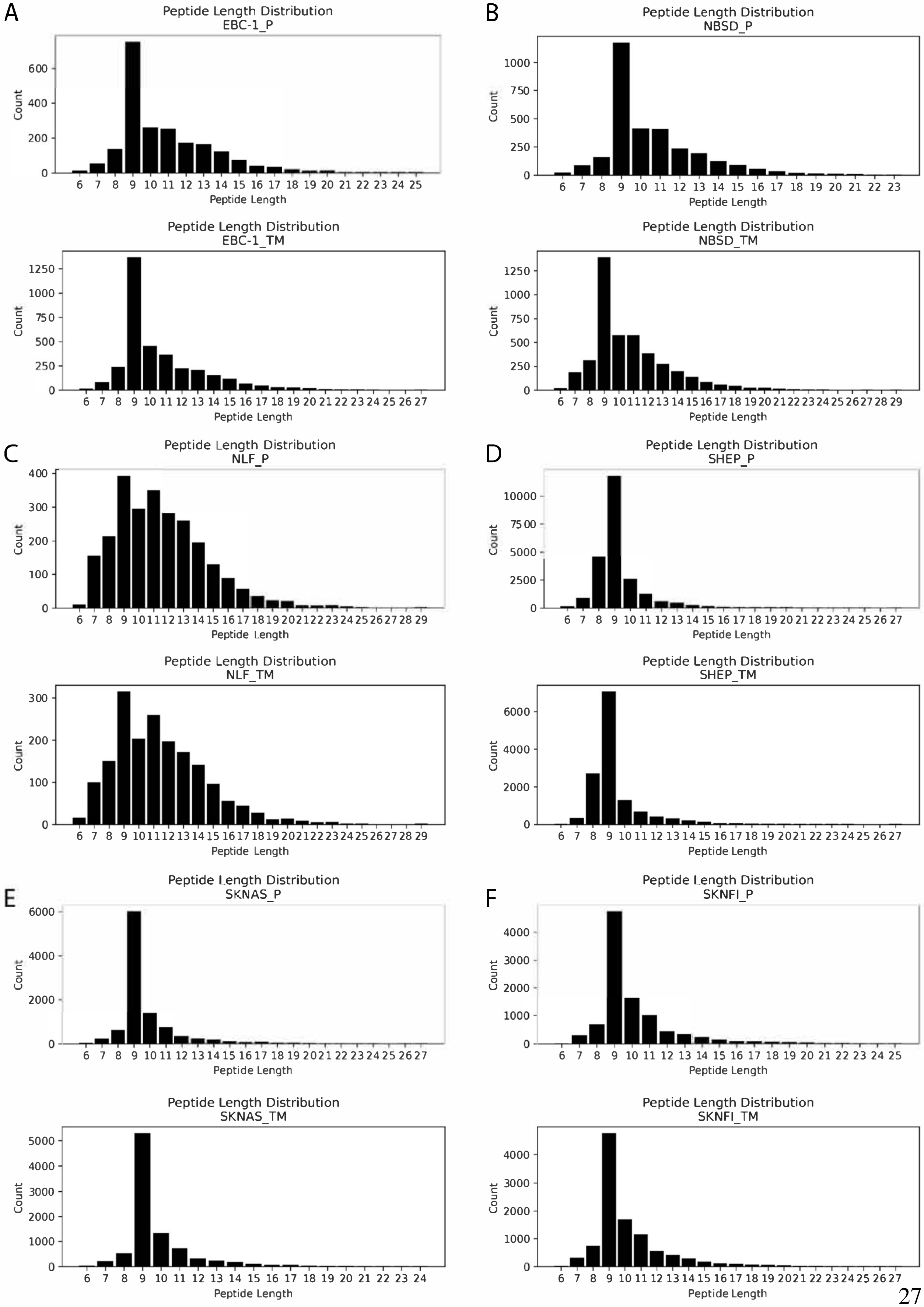
A
Peptide Length Distribution
EBC-1_P
Peptide Length Distribution
EBC-1_TM
B
Peptide Length Distribution
NBSD_P
Peptide Length Distribution
NBSD_TM
C
Peptide Length Distribution
NLF_P
Peptide Length Distribution
NLF_TM
D
Peptide Length Distribution
SHEP_P
Peptide Length Distribution
SHEP_TM
E
Peptide Length Distribution
SKNAS_P
Peptide Length Distribution
SKNAS_TM
F
Peptide Length Distribution
SKNFI_P
Peptide Length Distribution
SKNFI_TM
Count
Peptide Length

Supplemental Figure 11. Peptide length distribution of peptides derived from six neuroblastoma cell lines expressing tapasin-TM. Peptide distributions for **A)** EBc1**, B)** NBSD**, C)** NLF**, D)** SHEP**, E)** SKNAS and **F)** SKNFI. Parental cells are labeled as "P" and tapasin-TM expressing cells are labeled as "TM".

REFERENCES

1. K. Dhatchinamoorthy, J. D. Colbert, K. L. Rock, Cancer immune evasion through loss of MHC class I antigen presentation. *Front. Immunol.* **12**, 636568 (2021).

2. D. J. Zabransky, M. Yarchoan, E. M. Jaffee, Strategies for heating up cold tumors to boost immunotherapies. *Annu. Rev. Cancer Biol.* **7**, 149–170 (2023).

3. C. A. Klebanoff, S. S. Chandran, B. M. Baker, S. A. Quezada, A. Ribas, T cell receptor therapeutics: Immunological targeting of the intracellular cancer proteome. *Nat. Rev. Drug Discov.* **22**, 996–1017 (2023).

4. N. C. Colon, D. H. Chung, Neuroblastoma. *Adv. Pediatr.* **58**, 297–311 (2011).

5. M. L. Burr, C. E. Sparbier, K. L. Chan, Y. C. Chan, A. Kersbergen, E. Y. N. Lam, E. Azidis-Yates, D. Vassiliadis, C. C. Bell, O. Gilan, S. Jackson, L. Tan, S. Q. Wong, S. Hollizeck, E. M. Michalak, H. V. Siddle, M. T. McCabe, R. K. Prinjha, G. R. Guerra, B. J. Solomon, S. Sandhu, S. J. Dawson, P. A. Beavis, R. W. Tothill, C. Cullinane, P. J. Lehner, K. D. Sutherland, M. A. Dawson, An evolutionarily conserved function of polycomb silences the MHC class I antigen presentation pathway and enables immune evasion in cancer. *Cancer Cell* **36**, 385–401.e8 (2019).

6. M. Arnaud, J. Chiffelle, R. Genolet, B. Navarro Rodrigo, M. A. S. Perez, F. Huber, M. Magnin, T. Nguyen-Ngoc, P. Guillaume, P. Baumgaertner, C. Chong, B. J. Stevenson, D. Gfeller, M. Irving, D. E. Speiser, J. Schmidt, V. Zoete, L. E. Kandalaft, M. Bassani-Sternberg, S. Bobisse, G. Coukos, A. Harari, Sensitive identification of neoantigens and cognate TCRs in human solid tumors. *Nat. Biotechnol.* **40**, 656–660 (2022).

7. I. E. Shapiro, M. Bassani-Sternberg, The impact of immunopeptidomics: From basic research to clinical implementation. *Semin. Immunol.* **66**, 101727 (2023).

8. M. Yarmarkovich, Q. F. Marshall, J. M. Warrington, R. Premaratne, A. Farrel, D. Groff, W. Li, M. di Marco, E. Runbeck, H. Truong, J. S. Toor, S. Tripathi, S. Nguyen, H. Shen, T. Noel, N. L. Church, A. Weiner, N. Kendsersky, D. Martinez, R. Weisberg, M. Christie, L. Eisenlohr, K.

R. Bosse, D. S. Dimitrov, S. Stevanovic, N. G. Sgourakis, B. R. Kiefel, J. M. Maris, Targeting of intracellular oncoproteins with peptide-centric CARs. *Nature* **623**, 820–827 (2023).

9. Y. Sun, T. J. Florio, S. Gupta, M. C. Young, Q. F. Marshall, S. E. Garfinkle, G. F. Papadaki, H. V. Truong, E. Mycek, P. Li, A. Farrel, N. L. Church, S. Jabar, M. D. Beasley, B. R. Kiefel, M. Yarmarkovich, L. Mallik, J. M. Maris, N. G. Sgourakis, Structural principles of peptide-centric Chimeric Antigen Receptor recognition guide therapeutic expansion. *Sci. Immunol.* **8**, eadj5792 (2023).

10. R. L. Mayer, K. Mechtler, Immunopeptidomics in the era of single-cell proteomics. *Biology* **12**, 1514 (2023).

11. Y. Sykulev, M. Joo, I. Vturina, T. J. Tsomides, H. N. Eisen, Evidence that a single peptide-MHC complex on a target cell can elicit a cytolytic T cell response. *Immunity* **4**, 565–571 (1996).

12. P. Cresswell, A personal retrospective on the mechanisms of antigen processing. *Immunogenetics* **71**, 141–160 (2019).

13. Y. Shionoya, T. Kanaseki, S. Miyamoto, S. Tokita, A. Hongo, Y. Kikuchi, V. Kochin, K. Watanabe, R. Horibe, H. Saijo, T. Tsukahara, Y. Hirohashi, H. Takahashi, N. Sato, T. Torigoe, Loss of tapasin in human lung and colon cancer cells and escape from tumor-associated antigen-specific CTL recognition. *Oncoimmunology* **6**, e1274476 (2017).

14. E. Reeves, E. James, Antigen processing and immune regulation in the response to tumours. *Immunology* **150**, 16–24 (2017).

15. A. Tsherniak, F. Vazquez, P. G. Montgomery, B. A. Weir, G. Kryukov, G. S. Cowley, S. Gill, W. F. Harrington, S. Pantel, J. M. Krill-Burger, R. M. Meyers, L. Ali, A. Goodale, Y. Lee, G. Jiang, J. Hsiao, W. F. J. Gerath, S. Howell, E. Merkel, M. Ghandi, L. A. Garraway, D. E. Root, T. R. Golub, J. S. Boehm, W. C. Hahn, Defining a cancer dependency map. *Cell* **170**, 564–576.e16 (2017).

16. D. Ryan, S. Carberry, A. C. Murphy, A. U. Lindner, J. Fay, S. Hector, N. McCawley, O. Bacon, C. G. Concannon, E. W. Kay, D. A. McNamara, J. H. Prehn, Calnexin, an ER stress-

induced protein, is a prognostic marker and potential therapeutic target in colorectal cancer. *J. Transl. Med.* **14**, 196 (2016).

17. K. M. Paulsson, M. Jevon, J. W. Wang, S. Li, P. Wang, The double lysine motif of tapasin is a retrieval signal for retention of unstable MHC class I molecules in the endoplasmic reticulum. *J. Immunol.* **176**, 7482–7488 (2006).

18. A. C. McShan, C. A. Devlin, S. A. Overall, J. Park, J. S. Toor, D. Moschidi, D. Flores-Solis, H. Choi, S. Tripathi, E. Procko, N. G. Sgourakis, Molecular determinants of chaperone interactions on MHC-I for folding and antigen repertoire selection. *Proc. Natl. Acad. Sci. U.S.A.* **116**, 25602–25613 (2019).

19. M. L. van de Weijer, K. Samanta, N. Sergejevs, L. Jiang, M. E. Duenas, T. Heunis, T. Y. Huang, R. J. Kaufman, M. Trost, S. Sanyal, S. A. Cowley, P. Carvalho, Tapasin assembly surveillance by the RNF185/Membralin ubiquitin ligase complex regulates MHC-I surface expression. *Nat. Commun.* **15**, 8508 (2024).

20. C. Hermann, L. M. Strittmatter, J. E. Deane, L. H. Boyle, The binding of TAPBPR and Tapasin to MHC class I is mutually exclusive. *J. Immunol.* **191**, 5743–5750 (2013).

21. P. Parham, F. M. Brodsky, Partial purification and some properties of BB7.2. A cytotoxic monoclonal antibody with specificity for HLA-A2 and a variant of HLA-A28. *Hum. Immunol.* **3**, 277–299 (1981).

22. L. H. Boyle, C. Hermann, J. M. Boname, K. M. Porter, P. A. Patel, M. L. Burr, L. M. Duncan, M. E. Harbour, D. A. Rhodes, K. Skjodt, P. J. Lehner, J. Trowsdale, Tapasin-related protein TAPBPR is an additional component of the MHC class I presentation pathway. *Proc. Natl. Acad. Sci. U.S.A.* **110**, 3465–3470 (2013).

23. S. A. Overall, J. S. Toor, S. Hao, M. Yarmarkovich, M. O. R. Sara, G. I. Morozov, S. Nguyen, A. S. Japp, N. Gonzalez, D. Moschidi, M. R. Betts, J. M. Maris, P. Smibert, N. G. Sgourakis, High throughput pMHC-I tetramer library production using chaperone-mediated peptide exchange. *Nat. Commun.* **11**, 1909 (2020).

24. A. Neerincx, C. Hermann, R. Antrobus, A. van Hateren, H. Cao, N. Trautwein, S. Stevanovic, T. Elliott, J. E. Deane, L. H. Boyle, TAPBPR bridges UDP-glucose:glycoprotein glucosyltransferase 1 onto MHC class I to provide quality control in the antigen presentation pathway. *eLife* **6**, e23049 (2017).

25. A. Kaur, A. Surnilla, A. J. Zaitouna, M. B. Mumphrey, V. Basrur, I. Grigorova, M. Cieslik, M. Carrington, A. I. Nesvizhskii, M. Raghavan, Mass spectrometric profiling of HLA-B44 peptidomes provides evidence for tapasin-mediated tryptophan editing. *J. Immunol.* **211**, 1298–1307 (2023).

26. A. A. Bashirova, M. Viard, V. Naranbhai, A. Grifoni, W. Garcia-Beltran, M. Akdag, Y. Yuki, X. Gao, C. O'HUigin, M. Raghavan, S. Wolinsky, J. H. Bream, P. Duggal, J. Martinson, N. L. Michael, G. D. Kirk, S. P. Buchbinder, D. Haas, J. J. Goedert, S. G. Deeks, J. Fellay, B. Walker, P. Goulder, P. Cresswell, T. Elliott, A. Sette, J. Carlson, M. Carrington, HLA tapasin independence: Broader peptide repertoire and HIV control. *Proc. Natl. Acad. Sci. U.S.A.* **117**, 28232–28238 (2020).

27. J. G. Abelin, D. B. Keskin, S. Sarkizova, C. R. Hartigan, W. Zhang, J. Sidney, J. Stevens, W. Lane, G. L. Zhang, T. M. Eisenhaure, K. R. Clauser, N. Hacohen, M. S. Rooney, S. A. Carr, C. J. Wu, Mass spectrometry profiling of HLA-associated peptidomes in mono-allelic cells enables more accurate epitope prediction. *Immunity* **46**, 315–326 (2017).

28. D. R. Peaper, P. A. Wearsch, P. Cresswell, Tapasin and ERp57 form a stable disulfide-linked dimer within the MHC class I peptide-loading complex. *EMBO J.* **24**, 3613–3623 (2005).

29. F. Momburg, P. Tan, Tapasin-the keystone of the loading complex optimizing peptide binding by MHC class I molecules in the endoplasmic reticulum. *Mol. Immunol.* **39**, 217–233 (2002).

30. R. Jungmann, C. Steinhauer, M. Scheible, A. Kuzyk, P. Tinnefeld, F. C. Simmel, Single-molecule kinetics and super-resolution microscopy by fluorescence imaging of transient binding on DNA origami. *Nano Lett.* **10**, 4756–4761 (2010).

31. M. Dai, DNA-PAINT super-resolution imaging for nucleic acid nanostructures. *Methods Mol. Biol.* **1500**, 185–202 (2017).

32. Z. Liu, W. R. Legant, B. C. Chen, L. Li, J. B. Grimm, L. D. Lavis, E. Betzig, R. Tjian, 3D imaging of Sox2 enhancer clusters in embryonic stem cells. *eLife* **3**, e04236 (2014).

33. J. D. Colbert, F. M. Cruz, C. E. Baer, K. L. Rock, Tetraspanin-5-mediated MHC class I clustering is required for optimal CD8 T cell activation. *Proc. Natl. Acad. Sci. U.S.A.* **119**, e2122188119 (2022).

34. P. F. Robbins, R. A. Morgan, S. A. Feldman, J. C. Yang, R. M. Sherry, M. E. Dudley, J. R. Wunderlich, A. V. Nahvi, L. J. Helman, C. L. Mackall, U. S. Kammula, M. S. Hughes, N. P. Restifo, M. Raffeld, C. C. Lee, C. L. Levy, Y. F. Li, M. El-Gamil, S. L. Schwarz, C. Laurencot, S. A. Rosenberg, Tumor regression in patients with metastatic synovial cell sarcoma and melanoma using genetically engineered lymphocytes reactive with NY-ESO-1. *J. Clin. Oncol.* **29**, 917–924 (2011).

35. A. T. Kong, F. V. Leprevost, D. M. Avtonomov, D. Mellacheruvu, A. I. Nesvizhskii, MSFragger: Ultrafast and comprehensive peptide identification in mass spectrometry-based proteomics. *Nat. Methods* **14**, 513–520 (2017).

36. B. Reynisson, B. Alvarez, S. Paul, B. Peters, M. Nielsen, NetMHCpan-4.1 and NetMHCIIpan-4.0: Improved predictions of MHC antigen presentation by concurrent motif deconvolution and integration of MS MHC eluted ligand data. *Nucleic Acids Res.* **48**, W449–W454 (2020).

37. GTEx Consortium, The Genotype-Tissue Expression (GTEx) project. *Nat. Genet.* **45**, 580–585 (2013).

38. A. Marcu, L. Bichmann, L. Kuchenbecker, D. J. Kowalewski, L. K. Freudenmann, L. Backert, L. Muhlenbruch, A. Szolek, M. Lubke, P. Wagner, T. Engler, S. Matovina, J. Wang, M. Hauri-Hohl, R. Martin, K. Kapolou, J. S. Walz, J. Velz, H. Moch, L. Regli, M. Silginer, M. Weller, M. W. Loffler, F. Erhard, A. Schlosser, O. Kohlbacher, S. Stevanovic, H. G. Rammensee, M. C. Neidert, HLA Ligand Atlas: A benign reference of HLA-presented peptides to improve T-cell-based cancer immunotherapy. *J. Immunother. Cancer* **9**, e002071 (2021).

39. Z. Geng, E. Wafula, R. J. Corbett, Y. Zhang, R. Jin, K. S. Gaonkar, S. Shukla, K. S. Rathi, D. Hill, P. Wagner, A. Lahiri, D. P. Miller, A. Sickler, K. Keith, C. Blackden, A. Chroni, M. A. Brown, A. A. Kraya, K. L. Clark, B. R. Rood, A. C. Resnick, N. Van Kuren, J. M. Maris, A. Farrel, M. P. Koptyra, G. R. Trooskin, Y. Zhu, S. Stefankiewicz, Z. Abdullaev, A. T. Chinwalla, M. Santi, A. Q. Naqvi, J. L. Mason, C. J. Koschmann, X. Huang, S. J. Diskin, K. Aldape, B. K. Farrow, W. Ma, B. Zhang, E. B. Ennis, S. Tasian, S. Phul, M. R. Lueder, C. Zhong, J. M. Dybas, P. Wang, D. Taylor, T. Rokita, The Open Pediatric Cancer Project. *Gigascience* **14**, giaf093 (2025).

40. C. Nylund, P. Rappu, E. Pakula, A. Heino, L. Laato, L. L. Elo, P. Vihinen, S. Pyrhonen, G. R. Owen, H. Larjava, M. Kallajoki, J. Heino, Melanoma-associated cancer-testis antigen 16 (CT16) regulates the expression of apoptotic and antiapoptotic genes and promotes cell survival. *PLOS ONE* **7**, e45382 (2012).

41. J. L. Harenza, M. A. Diamond, R. N. Adams, M. M. Song, H. L. Davidson, L. S. Hart, M. H. Dent, P. Fortina, C. P. Reynolds, J. M. Maris, Transcriptomic profiling of 39 commonly-used neuroblastoma cell lines. *Sci. Data* **4**, 170033 (2017).

42. E. C. Jappe, C. Garde, S. H. Ramarathinam, E. Passantino, P. T. Illing, N. A. Mifsud, T. Trolle, J. V. Kringelum, N. P. Croft, A. W. Purcell, Thermostability profiling of MHC-bound peptides: A new dimension in immunopeptidomics and aid for immunotherapy design. *Nat. Commun.* **11**, 6305 (2020).

43. N. W. Mabe, M. Huang, G. N. Dalton, G. Alexe, D. A. Schaefer, A. C. Geraghty, A. L. Robichaud, A. S. Conway, D. Khalid, M. M. Mader, J. A. Belk, K. N. Ross, M. Sheffer, M. H. Linde, N. Ly, W. Yao, M. C. Rotiroti, B. A. H. Smith, M. Wernig, C. R. Bertozzi, M. Monje, C. S. Mitsiades, R. Majeti, A. T. Satpathy, K. Stegmaier, R. G. Majzner, Transition to a mesenchymal state in neuroblastoma confers resistance to anti-GD2 antibody via reduced expression of ST8SIA1. *Nat. Cancer* **3**, 976–993 (2022).

44. C. Chong, G. Coukos, M. Bassani-Sternberg, Identification of tumor antigens with immunopeptidomics. *Nat. Biotechnol.* **40**, 175–188 (2022).

45. N. M. Kendsersky, M. Odrobina, N. W. Mabe, A. Farrel, L. Grossmann, M. Tsang, D. Groff, A. J. Wolpaw, A. Narch, F. Zammarchi, P. H. van Berkel, C. V. Dang, Y. P. Mosse, K.

Stegmaier, J. M. Maris, Lineage dependence of the neuroblastoma surfaceome defines tumor cell state-dependent and -independent immunotherapeutic targets. *Neuro Oncol.* **27**, 1372–1384 (2025).

46. K. Pandey, S. S. Wang, N. A. Mifsud, P. Faridi, A. J. Davenport, A. I. Webb, J. J. Sandow, R. Ayala, M. Monje, R. S. Cross, S. H. Ramarathinam, M. R. Jenkins, A. W. Purcell, A combined immunopeptidomics, proteomics, and cell surface proteomics approach to identify immunotherapy targets for diffuse intrinsic pontine glioma. *Front. Oncol.* **13**, 1192448 (2023).

47. G. Kaur, S. Gras, J. I. Mobbs, J. P. Vivian, A. Cortes, T. Barber, S. B. Kuttikkatte, L. T. Jensen, K. E. Attfield, C. A. Dendrou, M. Carrington, G. McVean, A. W. Purcell, J. Rossjohn, L. Fugger, Structural and regulatory diversity shape HLA–C protein expression levels. *Nat. Commun.* **8**, 15924 (2017).

48. Y. Xu, R. Zou, J. Wang, Z.-W. Wang, X. Zhu, The role of the cancer testis antigen PRAME in tumorigenesis and immunotherapy in human cancer. *Cell Prolif.* **53**, e12770 (2020).

49. Q. Sun, J. Du, J. Dong, S. Pan, H. Jin, X. Han, J. Zhang, Systematic Investigation of the multifaceted role of *SOX11* in cancer. *Cancers* **14**, 6103 (2022).

50. M. Cardoso-Moreira, J. Halbert, D. Valloton, B. Velten, C. Chen, Y. Shao, A. Liechti, K. Ascencao, C. Rummel, S. Ovchinnikova, P. V. Mazin, I. Xenarios, K. Harshman, M. Mort, D. N. Cooper, C. Sandi, M. J. Soares, P. G. Ferreira, S. Afonso, M. Carneiro, J. M. A. Turner, J. L. VandeBerg, A. Fallahshahroudi, P. Jensen, R. Behr, S. Lisgo, S. Lindsay, P. Khaitovich, W. Huber, J. Baker, S. Anders, Y. E. Zhang, H. Kaessmann, Gene expression across mammalian organ development. *Nature* **571**, 505–509 (2019).

51. R. Vita, N. Blazeska, D. Marrama, I. C. T. Members, S. Duesing, J. Bennett, J. Greenbaum, M. De Almeida Mendes, J. Mahita, D. K. Wheeler, J. R. Cantrell, J. A. Overton, D. A. Natale, A. Sette, B. Peters, The Immune Epitope Database (IEDB): 2024 update. *Nucleic Acids Res.* **53**, D436–D443 (2025).

52. S. Lemke, M. L. Dubbelaar, P. Zimmermann, J. Bauer, A. Nelde, N. Hoenisch Gravel, J. Scheid, M. Wacker, S. Jung, A. Dengler, Y. Maringer, H. G. Rammensee, C. Gouttefangeas, S. Fillinger, T. Bilich, J. S. Heitmann, S. Nahnsen, J. S. Walz, PCI-DB: A novel primary tissue immunopeptidome database to guide next-generation peptide-based immunotherapy development. *J. Immunother. Cancer* **13**, e011366 (2025).

53. R. Bernards, S. K. Dessain, R. A. Weinberg, N-myc amplification causes down-modulation of MHC class I antigen expression in neuroblastoma. *Cell* **47**, 667–674 (1986).

54. W. L. Hwang, R. L. Wolfson, A. Niemierko, K. J. Marcus, S. G. DuBois, D. Haas-Kogan, Clinical impact of tumor mutational burden in neuroblastoma. *J. Natl. Cancer Inst.* **111**, 695–699 (2019).

55. C. H. Li, S. Sharma, A. A. Heczey, M. L. Woods, D. H. M. Steffin, C. U. Louis, B. J. Grilley, S. G. Thakkar, M. Wu, T. Wang, C. M. Rooney, M. K. Brenner, H. E. Heslop, Long-term outcomes of GD2-directed CAR-T cell therapy in patients with neuroblastoma. *Nat. Med.* **31**, 1125–1129 (2025).

56. S. Kumagai, Y. Momoi, H. Nishikawa, Immunogenomic cancer evolution: A framework to understand cancer immunosuppression. *Sci. Immunol.* **10**, eabo5570 (2025).

57. A. Blees, K. Reichel, S. Trowitzsch, O. Fisette, C. Bock, R. Abele, G. Hummer, L. V. Schafer, R. Tampe, Assembly of the MHC I peptide-loading complex determined by a conserved ionic lock-switch. *Sci. Rep.* **5**, 17341 (2015).

58. P. J. Lehner, M. J. Surman, P. Cresswell, Soluble tapasin restores MHC class I expression and function in the tapasin-negative cell line .220. *Immunity* **8**, 221–231 (1998).

59. B. H. Lan, M. Becker, C. Freund, The mode of action of tapasin on major histocompatibility class I (MHC-I) molecules. *J. Biol. Chem.* **299**, 102987 (2023).

60. G. Roder, L. Geironson, M. Rasmussen, M. Harndahl, S. Buus, K. Paulsson, Tapasin discriminates peptide-human leukocyte antigen-A*02:01 complexes formed with natural ligands. *J. Biol. Chem.* **286**, 20547–20557 (2011).

61. P. A. Wearsch, P. Cresswell, Selective loading of high-affinity peptides onto major histocompatibility complex class I molecules by the tapasin-ERp57 heterodimer. *Nat. Immunol.* **8**, 873–881 (2007).

62. I. K. Muller, C. Winter, C. Thomas, R. M. Spaapen, S. Trowitzsch, R. Tampe, Structure of an MHC I-tapasin-ERp57 editing complex defines chaperone promiscuity. *Nat. Commun.* **13**, 5383 (2022).

63. I. E. Shapiro, C. Maschke, J. Michaux, H. Pak, L. Wessling, T. Verkerk, R. Spaapen, M. Bassani-Sternberg, Deleterious KOs in the HLA class I antigen processing and presentation machinery induce distinct changes in the immunopeptidome. *Mol. Cell. Proteomics* **24**, 100951 (2025).

64. J. Anderson, R. G. Majzner, P. M. Sondel, Immunotherapy of neuroblastoma: Facts and hopes. *Clin. Cancer Res.* **28**, 3196–3206 (2022).

65. G. S. Reid, X. Shan, C. M. Coughlin, W. Lassoued, B. R. Pawel, L. H. Wexler, C. J. Thiele, M. Tsokos, J. L. Pinkus, G. S. Pinkus, S. A. Grupp, R. H. Vonderheide, Interferon-gamma-dependent infiltration of human T cells into neuroblastoma tumors in vivo. *Clin. Cancer Res.* **15**, 6602–6608 (2009).

66. A. Logan, "Development of targeted lipid nanoparticles for delivery of siRNA to neuroblastoma," thesis, UNSW Sydney (2023).

67. J. W. Schoggins, Interferon-stimulated genes: What do they all do? *Annu. Rev. Virol.* **6**, 567–584 (2019).

68. Z. Zou, Y. Hao, Z. Tao, W. Ye, Z. Luo, X. Li, R. Li, K. Zheng, J. Xia, C. Guo, X. Zhang, J. Wu, Current landscape of the immunoproteasome: Implications for disease and therapy. *Cell Death Discov.* **11**, 406 (2025).

69. G. R. Moe, L. M. Steirer, J. A. Lee, A. Shivakumar, A. D. Bolanos, A cancer-unique glycan: de-*N*-acetyl polysialic acid (dPSA) linked to cell surface nucleolin depends on re-expression of the fetal polysialyltransferase *ST8SIA2* gene. *J. Exp. Clin. Cancer Res.* **40**, 293 (2021).

70. M. Uhlen, C. Zhang, S. Lee, E. Sjostedt, L. Fagerberg, G. Bidkhori, R. Benfeitas, M. Arif, Z. Liu, F. Edfors, K. Sanli, K. von Feilitzen, P. Oksvold, E. Lundberg, S. Hober, P. Nilsson, J. Mattsson, J. M. Schwenk, H. Brunnstrom, B. Glimelius, T. Sjoblom, P. H. Edqvist, D. Djureinovic, P. Micke, C. Lindskog, A. Mardinoglu, F. Ponten, A pathology atlas of the human cancer transcriptome. *Science* **357**, eaan2507 (2017).

71. R. Barquera, E. Collen, D. Di, S. Buhler, J. Teixeira, B. Llamas, J. M. Nunes, A. Sanchez-Mazas, Binding affinities of 438 HLA proteins to complete proteomes of seven pandemic viruses and distributions of strongest and weakest HLA peptide binders in populations worldwide. *HLA* **96**, 277–298 (2020).

72. R. Darley, P. T. Illing, P. Duriez, A. Bailey, A. W. Purcell, A. van Hateren, T. Elliott, Evidence of focusing the MHC class I immunopeptidome by tapasin. *Front. Immunol.* **16**, 1563789 (2025).

73. T. Kuhn, J. Hettich, R. Davtyan, J. C. M. Gebhardt, Single molecule tracking and analysis framework including theory-predicted parameter settings. *Sci. Rep.* **11**, 9465 (2021).

74. A. Heckert, L. Dahal, R. Tjian, X. Darzacq, Recovering mixtures of fast-diffusing states from short single-particle trajectories. *eLife* **11**, e70169 (2022).

75. F. Yu, S. E. Haynes, A. I. Nesvizhskii, IonQuant enables accurate and sensitive label-free quantification with FDR-controlled match-between-runs. *Mol. Cell. Proteomics* **20**, 100077 (2021).

76. R. Arafeh, T. Shibue, J. M. Dempster, W. C. Hahn, F. Vazquez, The present and future of the cancer dependency map. *Nat. Rev. Cancer* **25**, 59–73 (2025).

77. B. M. Carreno, T. H. Hansen, Exogenous peptide ligand influences the expression and half-life of free HLA class I heavy chains ubiquitously detected at the cell surface. *Eur. J. Immunol.* **24**, 1285–1292 (1994).

78. V. Demichev, C. B. Messner, S. I. Vernardis, K. S. Lilley, M. Ralser, DIA-NN: Neural networks and interference correction enable deep proteome coverage in high throughput. *Nat. Methods* **17**, 41–44 (2020).

79. B. M. Carreno, M. Becker-Hapak, A. Huang, M. Chan, A. Alyasiry, W. R. Lie, R. L. Aft, L. A. Cornelius, K. M. Trinkaus, G. P. Linette, IL-12p70-producing patient DC vaccine elicits Tc1-polarized immunity. *J. Clin. Invest.* **123**, 3383–3394 (2013).
